# Optimal scaling of random walk Metropolis algorithms using Bayesian large-sample asymptotics

**DOI:** 10.1007/s11222-022-10080-8

**Published:** 2022-02-18

**Authors:** Sebastian M. Schmon, Philippe Gagnon

**Affiliations:** 1 Improbable, London, UK; 2 Durham University, Durham, UK; 3 Université de Montréal, Montréal, Canada

**Keywords:** Bernstein–von Mises theorem, Large-sample theory, Markov chain Monte Carlo, Optimal tuning, Weak convergence

## Abstract

High-dimensional limit theorems have been shown useful to derive tuning rules for finding the optimal scaling in random walk Metropolis algorithms. The assumptions under which weak convergence results are proved are, however, restrictive: the target density is typically assumed to be of a product form. Users may thus doubt the validity of such tuning rules in practical applications. In this paper, we shed some light on optimal scaling problems from a different perspective, namely a large-sample one. This allows to prove weak convergence results under realistic assumptions and to propose novel parameter-dimension-dependent tuning guidelines. The proposed guidelines are consistent with the previous ones when the target density is close to having a product form, and the results highlight that the correlation structure has to be accounted for to avoid performance deterioration if that is not the case, while justifying the use of a natural (asymptotically exact) approximation to the correlation matrix that can be employed for the very first algorithm run.

## Introduction

### Random walk Metropolis algorithms

Consider a Bayesian statistical framework where one wants to sample from an intractable posterior distribution $\pi$ to perform inference. This posterior distribution, also called the *target distribution* in a sampling context, is considered here to be that of model parameters $\varvec{\theta }\in \varvec{\varTheta }= {{\,\mathrm{\mathbb {R}}\,}}^d$, given a data sample of size *n*. We assume that $\pi$ has a probability density function (PDF) with respect to the Lebesgue measure; to simplify, we will also use $\pi$ to denote this density function. Tools called *random walk Metropolis (RWM)* algorithms (Metropolis et al. 1953), which are Markov chain Monte Carlo (MCMC) methods, can be employed to sample from $\pi$. An iteration of such an algorithm can be outlined as follows: given a current value of the chain $\varvec{\theta }$, a proposal for the next one is made using

$$\begin{aligned} \varvec{\theta }' := \varvec{\theta }+ \mathbf {S} \, \varvec{\epsilon }, \quad \varvec{\epsilon }\sim \varphi (\,\cdot \, ; \mathbf {0}, \mathbf {1}), \end{aligned}$$

where $\mathbf {S}$ is a scaling matrix and $\varphi (\,\cdot \, ; \mathbf {0}, \mathbf {1})$ denotes the standard normal distribution; this proposal is accepted with probability

$$\begin{aligned} \alpha (\varvec{\theta }, \varvec{\theta }') := \min \left\{ 1, \frac{\pi (\varvec{\theta }')}{\pi (\varvec{\theta })}\right\} ; \end{aligned}$$

if the proposal is rejected, the chain remains at the same state.

### Optimal scaling problems

Often, $\mathbf {S} = \lambda \mathbf {1}$, where $\lambda$ is a positive constant to be determined. In this case, $\lambda$ is the only free parameter. Yet, this parameter has to be tuned carefully because small values lead to tiny movements of the Markov chain simulated by RWM, while large values induce high rejection rates, both being undesirable. Finding the optimal value is thus a non-trivial problem. The last 20 years have witnessed a significant progress in the line of research studying such problems called *optimal scaling problems*, whether it is in RWM (Roberts et al. 1997; Bédard 2007; Sherlock and Roberts 2009; Durmus et al. 2017; Yang et al. 2020) or other algorithms including a scaling parameter (Roberts and Rosenthal 1998; Bédard et al. 2012; Beskos et al. 2013). In all these articles, the authors derive tuning rules based on analyses in the high-dimensional regime $d \rightarrow \infty$.

In the seminal work of Roberts et al. (1997) on RWM, the tuning rule for $\lambda$ follows from the analysis of a Langevin diffusion which is the limiting process of a re-scaled continuous-time version of RWM. The rule is remarkably simple: set $\lambda = \ell /\surd {d}$ and tune $\ell$ so that the acceptance rate is 0.234. The resulting optimal value is *universal*, in the sense that it minimizes the stationary integrated autocorrelation time of *any* function of the limiting process. The tuning rule is, however, derived under the assumption that $\pi (\varvec{\theta }) = \prod _{i = 1}^d f(\theta _i)$, where $\varvec{\theta }:= (\theta _1, \ldots , \theta _d)$ and *f* satisfies some regularity conditions. Assuming independent and identically distributed (IID) parameters considerably reduces the scope of applicability. One may be tempted to search for transformations/standardizations yielding IID parameters to expand the scope, but they exist only in specific situations (e.g. Gaussian target distributions). It will be seen that one of the main contributions of this paper is to provide formal and realistic conditions under which RWM algorithms targeting $\pi$ behave similarly to RWM targeting a Gaussian distribution with specific mean and covariance in an asymptotic regime. Our results thus allow to demonstrate that standardizing the parameters to expand the scope of applicability of the results of Roberts et al. (1997) is valid under regularity conditions, but only asymptotically.

The scope has been expanded otherwise in the past. For example, Bédard (2007) and Durmus et al. (2017) proved that the result is robust to departure from the *identically distributed* part of the assumption. Yang et al. (2020) proved that the result is valid under assumptions that are more general but difficult to verify. Empirical results in realistic scenarios where the IID assumption is, thus, not satisfied show that an acceptance rate of 0.234 is close to being optimal in these scenarios (e.g. Shang et al. 2015; Zhang et al. 2016; Gagnon et al. 2021), which can be seen as another demonstration of the robustness of the original results.

### Contributions

In this paper, we provide an alternative explanation of these empirical results in realistic scenarios, based on Bayesian large-sample theory. To achieve this, we revisit optimal scaling problems in RWM by exploiting important results underpinning that theory. In particular, we prove a weak convergence result as $n \rightarrow \infty$, with *d* being fixed, and derive tuning rules from it. While this asymptotic regime is ubiquitous in statistics, it is only recently that it was found useful in the analysis of MCMC algorithms (Deligiannidis et al. 2018; Gagnon 2021; Schmon et al. 2021a). Intuitively, if *n* is large enough and $\pi$ is a posterior distribution resulting from a sufficiently regular Bayesian model, then $\pi$ is close to a concentrating Gaussian, implying that RWM algorithms targeting $\pi$ behave like those targeting a Gaussian. This idea is formalized in Sect. 2.

The proximity between $\pi$ and a concentrating Gaussian can be established by virtue of Bernstein–von Mises theorems (see, e.g. Theorem 10.1 in Van der Vaart 2000 and Kleijn and Van der Vaart 2012). Verifying that a Bayesian model is sufficiently regular is thus closely related to verifying that the assumptions of such theorems are satisfied and has a priori nothing to do with whether the parameters are IID or not. Instead, such theorems rely on *local asymptotic normality*, meaning that a certain function of the $\log$-likelihood allows for a quadratic expansion (usually) around some “true” parameter value ${\varvec{\theta }_0}$. If the posterior concentrates around ${\varvec{\theta }_0}$, the quadratic expansion of the $\log$-likelihood implies an asymptotically Gaussian posterior; this happens under weak conditions such as IID *data points* with regularity conditions on the distribution and positive prior mass around ${\varvec{\theta }_0}$. The results in Roberts et al. (1997) actually rely on a similar quadratic expansion, but one that requires to impose a IID constraint on the parameters instead. We discuss in more detail the resemblance between both expansions in Sect. 3, allowing to establish a connection between our guidelines and theirs.

An advantage of the approach adopted in this paper to analyse MCMC algorithms is that a lot is known about which models are sufficiently regular (e.g. LeCam 1953; Bickel and Yahav 1969; Johnson 1970; Ghosal et al. 1995; Van der Vaart 2000; Kleijn and Van der Vaart 2012). Many models based on the exponential family are, for instance, regular enough. A notable example of such a model, namely Bayesian logistic regression, is studied in Sect. 4.

We finish this section by outlining our main contributions:

(i) presentation of a large-sample asymptotic framework and realistic assumptions under which a weak convergence of RWM is proved (Sect. 2);
(ii) an extensive analysis of the limiting RWM algorithm (Sect. 3) that allows to: (a) provide *dimension-dependent* optimal tuning guidelines, (b) show that the “0.234” rule-of-thumb is asymptotically valid from the point of view adopted in this paper in certain situations and that this rule is in fact quite robust to a departure from the IID assumption when $\mathbf {S} = \lambda \mathbf {1}$, without providing any guarantee regarding the algorithm performance; the latter deteriorates when there is a significant departure from the IID assumption and $\mathbf {S} = \lambda \mathbf {1}$ because this scaling matrix does not account for the correlation in between the parameters (Sect. 3);
(iii) justification of the use of natural asymptotically exact approximations to the covariance matrix such as the inverse Fisher information or its observed version that can be employed for the very first algorithm run to avoid deterioration of performance.

Our analysis is mainly based on an efficiency measure called the *expected squared jumping distance (ESJD)*. It is defined as the average squared distance between two consecutive states (or a function of them). Optimizing this measure does *not* yield a universally optimal scaling because it is optimal for *one* function and thus not necessarily for *all* functions. Typically, $\mathrm {ESJD}$ is optimized for the identity function; this strategy has demonstrated on many occasions in the literature to lead to reliable conclusions (see, e.g., Yang et al. (2020)). This choice also allows to establish a formal connection between our results and those of Roberts et al. (1997) in Sect. 3.

### Notation and framework

We first note that within our framework the Bayesian posterior $\pi$ depends on *n*; therefore, from now on the target will be denoted by $\pi _n$. The target being a posterior distribution in fact depends on a set of observations that will be denoted by $\mathbf {y}_{1:n} := (\mathbf {y}_1, \ldots , \mathbf {y}_n) \in \prod _{i=1}^n \varvec{\mathsf {Y}}_i$. We make this dependence implicit to simplify. We assume $\mathbf {y}_{1:n}$ to be the first *n* components of a realization of some unknown data generating process $\mathbb {P}^{\mathbf {Y}}$ on $\prod _{i=1}^\infty \varvec{\mathsf {Y}}_i$. Through its dependence on the data points, the distribution $\pi _n$ is a random measure on $\mathbb {R}^d$. Consequently, everything derived from it (or in fact directly from the data points) is random, such as integrals with respect to $\pi _n$ and the distributions of Markov chains produced by RWM targeting $\pi _n$. In the following, we make statements about the convergence of such mathematical objects in $\mathbb {P}^{\mathbf {Y}}$-probability. We now briefly describe what we mean by this and refer to Schmon (2020) and Schmon et al. (2021b, Section S1) for more details on random measures and such convergences in a MCMC context. We say, for instance, that an integral with respect to $\pi _n$, denoted by $I_n$, converges to *I* in $\mathbb {P}^{\mathbf {Y}}$-probability when $\mathbb {P}^{\mathbf {Y}}|I_n - I| \rightarrow 0$. A Markov chain produced by RWM targeting $\pi _n$ is seen to weakly converge in $\mathbb {P}^{\mathbf {Y}}$-probability towards another Markov chain when the finite-dimensional distributions converge in $\mathbb {P}^{\mathbf {Y}}$-probability, where the latter can be seen as random integrals involving $\pi _n$ and random transition kernels.

The matrix $\mathbf {S}$ will also depend on *n* and will thus be written $\mathbf {S}_n$. We use $\varphi (\varvec{\theta }; \varvec{\mu }, \varvec{\varSigma })$ to denote a Gaussian density with argument $\varvec{\theta }$, mean $\varvec{\mu }$, and covariance matrix $\varvec{\varSigma }$ and use $\varPhi$ to denote the cumulative distribution function of a standard normal; $\mathcal {I}{(\varvec{\theta })}$ and $\hat{\varvec{\theta }}_n$ denote the Fisher information evaluated at $\varvec{\theta }$ and a parameter estimator, respectively. Finally, the norm of a vector $\varvec{\mu }$ with respect to a matrix ${\varvec{\varSigma }}$ is denoted by $\Vert {\varvec{\mu }}\Vert _{{\varvec{\varSigma }}}^2 := {\varvec{\mu }}^T {\varvec{\varSigma }}{\varvec{\mu }}$. We simply write $\Vert \varvec{\mu }\Vert ^2$ when ${\varvec{\varSigma }} = \mathbf {1}$.

## Large-sample asymptotics of RWM

We first present three conditions under which a weak convergence of RWM can be established, and next, our result. The first condition is that a Bernstein–von Mises theorem holds, i.e. the concentration of the PDF $\pi _n$ around the true model parameter value $\varvec{\theta }_0$, as *n* increases, with a shape that resembles that of a Gaussian. For simplicity, we only consider the case where the Bayesian model is well specified, but our result remains valid under model misspecification; however, in this case, ${\varvec{\theta }}_0$ is some fixed parameter value and the covariance matrix of the Gaussian is different (see Kleijn and Van der Vaart 2012).

### Assumption 1

(*Bernstein–von Mises theorem*) As $n \rightarrow \infty$, we have the following convergences in $\mathbb {P}^{\mathbf {Y}}$-probability:

$$\begin{aligned}&\int \left| {\pi }_{n}(\varvec{\theta })-\varphi (\varvec{\theta }; \hat{\varvec{\theta }}_{n}, \mathcal {I}{(\varvec{\theta }_0)}^{-1}/n)\right| \mathrm {d}\varvec{\theta }\rightarrow 0 \\&\text {with} \quad \hat{\varvec{\theta }}_n \rightarrow \varvec{\theta }_0. \end{aligned}$$

If the posterior concentrates at a rate of $1/\surd {n}$, the scaling of the random walk needs to decrease at the same rate. Note that this is an analogous requirement to that in Roberts et al. (1997); in that paper, the scaling diminishes with *d* like $1/\surd {d}$. In both cases, it is to accommodate to the reality that, as *n* or *d* increases, the acceptance rate rapidly deteriorates if the scaling is not suitably reduced.

The scaling matrix is more precisely considered here to be of the following form: $\mathbf {S}_n = (\lambda / \surd {n}) \mathbf {M}_n$, with $\mathbf {M}_n$ a matrix that is allowed to depend on *n* (and the data, but this dependence is made implicit to simplify the notation). The second assumption is now presented.

### Assumption 2

(*Proposal scaling*) The proposal is scaled as follows: $\mathbf {S}_n = (\lambda / \surd {n}) \mathbf {M}_n$, and there exists a matrix $\mathbf {M}$ such that $\mathbf {M}_n\mathbf {M}_n^T \rightarrow \mathbf {M}\mathbf {M}^T$ in $\mathbb {P}^{\mathbf {Y}}$-probability, where we say that a matrix converges in probability whenever all entries converge in probability.

A choice of matrix $\mathbf {M}_n$ that satisfies Assumption 2 is the identity matrix $\mathbf {1}$. In the following, it will be seen that choosing $\mathbf {M}_n$ to be the result of a Cholesky decomposition of $\mathcal {I}{(\hat{\varvec{\theta }}_n)}^{-1}$, i.e. such that $\mathbf {M}_n \mathbf {M}_n^T = \mathcal {I}{(\hat{\varvec{\theta }}_n)}^{-1}$, may be preferable, depending on the strength of the correlation between the parameters. When the correlation is significant, the desirable property is that $\mathbf {M}_n \mathbf {M}_n^T \rightarrow \mathbf {M}\mathbf {M}^T = \mathcal {I}{(\varvec{\theta }_0)}^{-1}$ in $\mathbb {P}^\mathbf {Y}$-probability, which is often the case for regular models when $\mathbf {M}_n \mathbf {M}_n^T = \mathcal {I}{(\hat{\varvec{\theta }}_n)}^{-1}$. Note that other choices of matrices $\mathbf {M}_n$ may have this property. For instance, it may be valid to choose $\mathbf {M}_n$ to be the result of a Cholesky decomposition of the inverse observed information matrix instead.

Given that the target distribution concentrates and the proposal scaling decreases, we need to standardize the Markov chains simulated by RWM to obtain a non-trivial limit. For each time step, we consider the transformation $\mathbf {z}_n := n^{1/2}(\varvec{\theta }_n - \hat{\varvec{\theta }}_n)$. The proposals after the transformation are thus $\mathbf {z}_n' = \mathbf {z}_n + \lambda \mathbf {M}_n \varvec{\epsilon }$ and the resulting Markov chains have a stationary PDF $\pi _{\mathbf {Z}_n}$ which is such that $\pi _{\mathbf {Z}_n}(\mathbf {z}_n) = \pi _n(\hat{\varvec{\theta }}_n + n^{-1/2}\mathbf {z}_n)/n^{d/2}$. This implies that the proposals are sampled from a Gaussian with a non-decreasing scaling and the stationary distribution behaves like a Gaussian with mean $\mathbf {0}$ and covariance $\mathcal {I}{(\varvec{\theta }_0)}^{-1}$, as $n \rightarrow \infty$. Let $\varXi _{n} := \big (\mathbf {Z}_{k, n}\big )_{k\geqslant 0}$ be such a standardized Markov chain with $\mathbf {Z}_{k, n}$ being the state of the chain after *k* iterations.

An asymptotic result that we prove is a convergence of $\varXi _{n}$ towards $\varXi := \big (\mathbf {Z}_{k}\big )_{k\geqslant 0}$, which is a Markov chain simulated by a RWM algorithm targeting a Gaussian with mean $\mathbf {0}$ and covariance $\mathcal {I}{(\varvec{\theta }_0)}^{-1}$ using proposals given by $\mathbf {z}' = \mathbf {z} + \lambda \mathbf {M} \varvec{\epsilon }$.

To obtain the result, we assume that the chains start in stationarity. If this is not the case, the result generally still holds (at least approximatively), but for subchains formed of states with iteration indices larger than a certain threshold. Indeed, the chains produced by RWM are irreducible and they are typically aperiodic (they are if there are positive probabilities of rejecting proposals); therefore, they are typically ergodic (Tierney 1994). This implies that the chains typically reach stationarity (at least approximatively) after a large enough number of iterations.

### Assumption 3

(*Stationarity*) $\varXi _{n}$ and $\varXi$ start in stationarity.

We are now ready to present the main theoretical results of this paper.

### Theorem 1

Under Assumptions 1, 2 and 3, we have the following convergences in $\mathbb {P}^\mathbf {Y}$-probability:

(i) $\varXi _{n}$ converges weakly to $\varXi$;
(ii) the expected acceptance probability converges,
  $$\begin{aligned}&\mathbb {E}\left[ \min \left\{ 1, \frac{\pi _{\mathbf {Z}_n}(\mathbf {Z}_n')}{\pi _{\mathbf {Z}_n}(\mathbf {Z}_n)}\right\} \right] \\&\quad \rightarrow \mathbb {E}\left[ \min \left\{ 1, \frac{\varphi (\mathbf {Z}'; \mathbf {0}, \mathcal {I}{(\varvec{\theta }_0)}^{-1})}{\varphi (\mathbf {Z}; \mathbf {0}, \mathcal {I}{(\varvec{\theta }_0)}^{-1})}\right\} \right] , \end{aligned}$$
  with
  $$\begin{aligned}&\mathbf {Z}_n \sim \pi _{\mathbf {Z}_n},&\mathbf {Z}_n' \sim \varphi (\, \cdot \,; \mathbf {Z}_n, \lambda ^2 \mathbf {M}_n \mathbf {M}_n^T), \\&\mathbf {Z} \sim \varphi (\, \cdot \,; \mathbf {0}, \mathcal {I}{(\varvec{\theta }_0)}^{-1}),&\mathbf {Z}' \sim \varphi (\, \cdot \,; \mathbf {Z}, \lambda ^2 \mathbf {M} \mathbf {M}^T); \end{aligned}$$
(iii) if additionally
  $$\begin{aligned} \mathbf {M}_n \mathbf {M}_n^T = \mathcal {I}{(\hat{\varvec{\theta }}_n)}^{-1} \rightarrow \mathbf {M}\mathbf {M}^T = \mathcal {I}{(\varvec{\theta }_0)}^{-1} \end{aligned}$$
  in $\mathbb {P}^\mathbf {Y}$-probability, then the ESJD converges,
  $$\begin{aligned} \mathbb {E}\left[ \Vert \mathbf {Z}_{k + 1, n} - \mathbf {Z}_{k, n}\Vert _{\mathcal {I}{(\hat{\varvec{\theta }}_n)}}^2 \right] \rightarrow \mathbb {E}\left[ \Vert \mathbf {Z}_{k + 1} - \mathbf {Z}_k\Vert _{\mathcal {I}{(\varvec{\theta }_0)}}^2 \right] . \end{aligned}$$

The proof of Theorem 1 and the proof of all the following theoretical results are deferred to Appendix A. Note that, as shown in the proof, Result (iii) holds under a more general, but more technical, assumption.

## Tuning guidelines and analysis of the limiting RWM

We first present in Sect. 3.1 special cases of the limiting ESJD resulting from specific choices for $\mathbf {M}$; these special cases will be seen to suggest tuning guidelines. Subsequently, we turn to an extensive analysis of the limiting RWM in Sect. 3.2 showing the relevance of these guidelines, but also the robustness of the 0.234 rule when $\mathbf {M} = \mathbf {1}$. An interesting feature of the proposed guidelines is that they are consistent with this rule. An asymptotic connection with the results of Roberts et al. (1997) as $d \rightarrow \infty$ is established in Sect. 3.3.

### Tuning guidelines

In the same spirit as Roberts et al. (1997) who optimize the speed measure of their limiting diffusion as a proxy, we propose here to optimize

$$\begin{aligned} \mathbb {E}\left[ \Vert \mathbf {Z}_{k + 1} - \mathbf {Z}_k\Vert _{\mathcal {I}{({\varvec{\theta }}_0)}}^2 \right] =: \mathrm {ESJD}(\lambda , \mathbf {M})\end{aligned}$$

with respect to the tuning parameter $\lambda$, for given $\mathbf {M}$. There exists a simple expression for $\mathrm {ESJD}(\lambda , \mathbf {M})$ for the typical choice $\mathbf {M} = \mathbf {1}$ or when $\mathbf {M}$ results from a Cholesky decomposition of $\mathcal {I}{(\varvec{\theta }_0)}^{-1}$, i.e. when $\mathbf {M} \mathbf {M}^T = \mathcal {I}{(\varvec{\theta }_0)}^{-1}$. The expressions are provided in Corollary 1, along with the expected acceptance probabilities associated with these special cases of $\mathbf {M}$.

#### Corollary 1

(Formulae for ESJD and acceptance probabilities) Assume $\varXi$ starts in stationarity and let $\varvec{\epsilon }\sim \varphi (\,\cdot \, ; \mathbf {0}, \mathbf {1})$. If $\mathbf {M} = \mathbf {1}$,

1 $$\begin{aligned} \mathrm {ESJD}(\lambda , \mathbf {M}) = 2 \lambda ^2 \, \mathbb {E}\left[ \Vert {\varvec{\epsilon }}\Vert _{\mathcal {I}{({\varvec{\theta }}_0)}}^2 \, \varPhi \left( -\lambda \, \frac{\Vert {\varvec{\epsilon }}\Vert _{\mathcal {I}{({\varvec{\theta }}_0)}}}{2}\right) \right] , \end{aligned}$$

and the expected acceptance probability is

$$\begin{aligned} 2 \, \mathbb {E}\left[ \, \varPhi \left( -\lambda \, \frac{\Vert \varvec{\epsilon }\Vert _{\mathcal {I}{(\varvec{\theta }_0)}}}{2}\right) \right] . \end{aligned}$$

If $$\mathbf {M} \mathbf {M}^T = \mathcal {I}{(\varvec{\theta }_0)}^{-1}$$,

2 $$\begin{aligned} \mathrm {ESJD}(\lambda , \mathbf {M}) = 2\lambda ^2 \, \mathbb {E}\left[ \Vert {\varvec{\epsilon }}\Vert ^2 \, \varPhi \left( -\lambda \, \frac{\Vert {\varvec{\epsilon }}\Vert }{2}\right) \right] \end{aligned}$$

and the expected acceptance probability is

$$\begin{aligned} 2 \, \mathbb {E}\left[ \, \varPhi \left( -\lambda \, \frac{\Vert \varvec{\epsilon }\Vert }{2}\right) \right] . \end{aligned}$$

In general, expressions (1) and (2) in Corollary 1 cannot be optimized analytically, but can be approximated efficiently using independent Monte Carlo sampling, and thus, numerically optimized using the resulting approximations. We note that (1) and (2) coincide when $\mathcal {I}{(\varvec{\theta }_0)} = \mathbf {1}$ and that, in general, (1) depends on $\mathcal {I}{(\varvec{\theta }_0)}$, while (2) does not. This reveals that the value of $\lambda$ maximizing (1) is similar to that maximizing (2) when the model parameters are close to be IID, but is expected to be different otherwise. More precisely, it is expected that the value of $\lambda$ maximizing (1) is small when the parameters are strongly correlated, yielding inefficient RWM algorithms; this is confirmed in Sect. 3.2. Corollary 1 also reveals that, when $\mathbf {M}$ is such that $\mathbf {M} \mathbf {M}^T = \mathcal {I}{(\varvec{\theta }_0)}^{-1}$, the optimal value for $\lambda$ is invariant to the covariance structure. In other words, Corollary 1 suggests the following practical guideline: *set *$\mathbf {S}_n = (\lambda / \surd {n}) \mathbf {M}_n$* with* $\mathbf {M}_n$* such that *$\mathbf {M}_n \mathbf {M}_n^T = \mathcal {I}{(\hat{\varvec{\theta }}_n)}^{-1}$. Aiming to match the proposal covariance to the target covariance has a long history in MCMC (see, e.g., Haario et al. (2001) in a context of adaptive algorithms). To exactly match the target covariance, $\mathbf {S}_n$ is typically set to $\mathbf {S}_n = (\lambda / \surd {n}) \mathbf {1}$ and trial runs are performed to estimate the covariance. This may turn out to be ineffective when RWM with this choice of scaling matrix performs poorly. The guideline proposed here provides an alternative: while the matrix used to build $\mathbf {S}_n$ *does not* correspond to the target covariance, it is *asymptotically equivalent* to it (under the assumptions mentioned in Sect. 2); the advantage is that this alternative can be implemented for the very first algorithm run.

In Table 1, we present the results of a numerical optimization of $\mathrm {ESJD}(\lambda , \mathbf {M})$ when $\lambda = \ell / \surd {d}$ and $\mathbf {M}$ is such that $\mathbf {M} \mathbf {M}^T = \mathcal {I}{(\varvec{\theta }_0)}^{-1}$ based on Monte Carlo samples of size 10,000,000 and a grid search, for several values of *d*. The optimization is thus with respect to $\ell$, and the optimal value is denoted by $\hat{\ell }$. Note that we have observed empirically that optimizing the effective sample size (ESS) yields similar results. Note also that the code to produce all numerical results is available online^1^. In Table 1, additionally to $\hat{\ell }$, we present the acceptance rate, i.e. the Monte Carlo estimate of the expected acceptance probability, of the RWM using $\hat{\ell }$. This table thus serves as guidelines to set $\ell$ in $\mathbf {S}_n = (\ell / \surd {d n}) \mathbf {M}_n$ with $\mathbf {M}_n$ such that $\mathbf {M}_n \mathbf {M}_n^T = \mathcal {I}{(\hat{\varvec{\theta }}_n)}^{-1}$. Writing $\lambda = \ell / \surd {d}$ allows to establish a connection with the results of Roberts et al. (1997) in Sect. 3.3. The existence of such a connection is highlighted by the values of the optimal acceptance rates for large values of *d*. In Sect. 3.3, we establish that ESJD converges as $d \rightarrow \infty$ to the same expression which is optimized in Roberts et al. (1997) and which leads within their framework to an optimal acceptance rate of $23.38\%$. From this result, we prove that the asymptotically optimal acceptance rate derived within our framework is $23.38\%$ as well. What is remarkable is that, not only do we retrieve within our framework the same value as Roberts et al. (1997) when the parameters are IID, i.e. when $\mathcal {I}{(\varvec{\theta }_0)}^{-1} = \mathbf {1}$, but the limiting optimal acceptance rate is also $23.38\%$ when $\mathcal {I}{(\varvec{\theta }_0)} \ne \mathbf {1}$, as long as $\mathbf {M} \mathbf {M}^T = \mathcal {I}{(\varvec{\theta }_0)}^{-1}$, which is a consequence of the invariance of (2), a quality that the acceptance rate also has.

From Table 1, we observe that when $\mathbf {M}$ is such that $\mathbf {M} \mathbf {M}^T = \mathcal {I}{(\varvec{\theta }_0)}^{-1}$, the optimal acceptance rate is approximately 44% for $d=1$, 35% for $d=2$ and decreases towards 23.38% as *d* increases, regardless of the covariance structure. A theoretical result allows to support our numerical findings. Proposition 1 states that, for fixed $\ell$, the expected acceptance probability decreases monotonically as *d* increases, which confirms, for instance, that from $d = 1$ to $d = 2$ with $\ell = \hat{\ell } = 2.42$ fixed, the expected acceptance probability decreases.

#### Proposition 1

Let $$\varvec{\epsilon }\sim \varphi (\,\cdot \, ; \mathbf {0}, \mathbf {1})$$. For $$d \ge 2$$,

$$\begin{aligned} 2 \, \mathbb {E}\left[ \, \varPhi \left( -\frac{\ell }{2}\sqrt{\frac{1}{d}\sum _{i = 1}^d \epsilon _i^2}\right) \right] \le 2 \, \mathbb {E}\left[ \, \varPhi \left( -\frac{\ell }{2}\sqrt{\frac{1}{d-1}\sum _{i = 1}^{d - 1} \epsilon _i^2}\right) \right] . \end{aligned}$$

We finish this section by noting that for $d = 1$, the ESJD and expected acceptance probability of a RWM targeting a Gaussian distribution have closed-form expressions (see Sherlock and Roberts 2009) and can thus be optimized using these expressions.

**Table 1:** Optimal value for $\ell$ and the acceptance rate of the limiting RWM using this value and $\mathbf {M}$ such that $\mathbf {M} \mathbf {M}^T = \mathcal {I}{(\varvec{\theta }_0)}^{-1}$, as a function of *d*

*d*	1	2	3	4	5	10	15	20	30	50
$\hat{\ell }$	2.42	2.42	2.42	2.42	2.40	2.40	2.39	2.39	2.38	2.38
Acc. rate. (in %)	44.00	35.00	31.30	29.29	28.39	25.78	25.07	24.61	24.34	23.97

### Analysis of the limiting RWM

We now present the practical implications of the guidelines proposed in Sect. 3.1 (in the asymptotic regime $n \rightarrow \infty$) through an analysis of the impact of different target covariances on the performance and acceptance rate of the optimal limiting RWM. More precisely, we analyse the behaviour of the limiting RWM with $\mathbf {M} = \mathbf {1}$ and $\mathbf {M}$ such that $\mathbf {M} \mathbf {M}^T = \mathcal {I}{(\varvec{\theta }_0)}^{-1}$ under different target covariances; for each of these covariances, the algorithms are made optimal, in the sense that $\lambda$ (or $\ell$) is tuned according to the expressions in Corollary 1 (or Table 1). The algorithm with $\mathbf {M}$ such that $\mathbf {M} \mathbf {M}^T = \mathcal {I}{(\varvec{\theta }_0)}^{-1}$ has a higher complexity because an additional matrix multiplication is required every iteration. However, in standard modern statistical computing frameworks we found both algorithms to take roughly the same amount of time to complete; it is the case for instance for the numerical experiments presented in this paper that were performed in R (R Core Team 2020) on a computer with an i9 CPU.

**Fig. 1:**
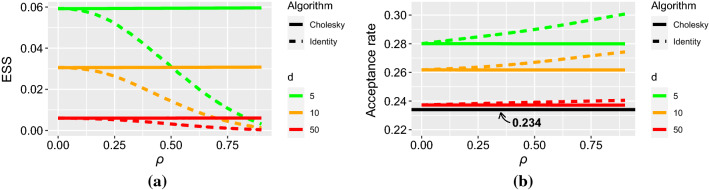
Optimal (**a**) ESS and (**b**) acceptance rate of the limiting RWM with $\mathbf {M} = \mathbf {1}$ and with $\mathbf {M}$ such that $\mathbf {M} \mathbf {M}^T = \mathcal {I}{(\varvec{\theta }_0)}^{-1}$ as a function of $\rho$ in the case where the (*i*, *j*)th entry of $\mathcal {I}{(\varvec{\theta }_0)}^{-1}$ is given by $\rho ^{|i-j|}$, when $d = 5, 10, 50$

For the analysis, we focus on showing what happens when the correlation between the model parameters increases under a specific covariance structure: the (*i*, *j*)th entry of $\mathcal {I}{(\varvec{\theta }_0)}^{-1}$ is given by $\rho ^{|i-j|}$, where $-1 \le \rho \le 1$ is a varying parameter. This covariance structure is often called *autoregressive of order 1* and represents a situation where the parameters are standardized, in the sense that their marginal variances are all equal to 1, and the correlations between them decline exponentially with distance, at a speed that depends on $\rho$. In this setting, the target covariance matrix is parametrized with only one parameter, $\rho$. The case where $0 \le \rho \le 1$ is more interesting for the current purpose; a value close to 0 leads to weak correlations between the parameters, whereas a value close to 1 makes the correlation persist with distance, yielding strong correlations between the parameters. Note that the situation where parameters are standardized and $\mathbf {M} = \mathbf {1}$ is equivalent to that where the parameters are non-standardized but $\mathbf {M}$ is a diagonal matrix with diagonal entries equal to the marginal standard deviations. The empirical results are presented in Fig. 1.

In Fig. 1, the algorithm performances are evaluated using the minimum of the marginal ESSs, reported per iteration. ESJD cannot be used to evaluate performance across different values of $\rho$ because using a norm with respect to $\mathcal {I}{(\varvec{\theta }_0)}$ in ESJD standardizes this measure. We show the results for $0 \le \rho \le 0.9$ as beyond 0.9, RWM with $\mathbf {M} = \mathbf {1}$ becomes unreliable. As suggested by the expressions in Corollary 1, the performance of RWM with $\mathbf {M}$ such that $\mathbf {M} \mathbf {M}^T = \mathcal {I}{(\varvec{\theta }_0)}^{-1}$ does not vary with $\rho$, while it does for RWM with $\mathbf {M} = \mathbf {1}$; it in fact deteriorates when $\rho$ increases due to an optimal value for $\ell$ that decreases. As for the acceptance rate, it is invariant as well for RWM with the Cholesky decomposition matrix and increases slightly with $\rho$ for RWM with the identity matrix. The optimal acceptance rate becomes closer to 0.234 as *d* increases when $\rho = 0$, which is not surprising given that the target in this case satisfies the assumptions of Roberts et al. (1997). It is, however, remarkable that, for $\mathbf {M} = \mathbf {1}$, the optimal acceptance rate only slightly increases as $\rho$ gets closer to 1.

### Connection to scaling limits

The aim of this section is to establish a formal connection between our guidelines and those of Roberts et al. (1997) through an asymptotic analysis of features of the limiting chain $\varXi := \big (\mathbf {Z}_{k}\big )_{k\geqslant 0}$ as *d* increases. In particular, it will be pointed out using a theoretical argument that our guidelines are consistent in that we find equivalent asymptotically optimal values for $\ell$ and acceptance rate as these authors. The stationary distribution of $\varXi$, which is a Gaussian with mean $\mathbf {0}$ and covariance $\mathcal {I}{(\varvec{\theta }_0)}^{-1}$, can be seen as a special case of the product target studied by Roberts et al. (1997) when $\mathcal {I}{(\varvec{\theta }_0)}^{-1} = \mathbf {1}$. As mentioned in the previous sections, it is thus not surprising but reassuring to find the same asymptotically optimal values within our framework for this special case.

To find the optimal values for RWM in the high-dimensional limit, we analyse the expected acceptance probability and $\mathrm {ESJD}(\lambda , \mathbf {M})$ by considering them as sequences indexed by *d*, and let $d \rightarrow \infty$. We provide a result establishing that $\mathrm {ESJD}(\lambda , \mathbf {M})$ converges towards a function that is equivalent to that optimized in Roberts et al. (1997), when $\lambda = \ell /\surd {d}$ and the proposal covariance is set to $\mathbf {M} \mathbf {M}^T = \mathcal {I}{(\varvec{\theta }_0)}^{-1}$. The ESJD is optimized by an equivalent value for $\ell$, and the expected acceptance probability converges to the same limiting acceptance rate as Roberts et al. (1997), which is seen to imply that the asymptotically optimal acceptance rate is the same. The asymptotically optimal values are 2.38 and 0.234 for $\ell$ and the acceptance rate, respectively. Within our framework, these values are optimal for any target covariance $\mathcal {I}{(\varvec{\theta }_0)}^{-1}$ given that the limiting acceptance rate and $\mathrm {ESJD}$ do not depend on $\mathcal {I}{(\varvec{\theta }_0)}^{-1}$.

Before presenting the formal results, we provide an informal argument explaining why the connection exists and more precisely why $\mathrm {ESJD}(\lambda , \mathbf {M})$ converges towards a function that is equivalent to that in Roberts et al. (1997). Central to the reason why the efficiency measures are asymptotically the same are the convergences of the acceptance rates in both contexts to a constant as $d \rightarrow \infty$. To provide the informal argument, we thus present the acceptance rates and show how Taylor expansions explain their asymptotic behaviour. We start with that in Roberts et al. (1997); we thus consider a sequence of target densities $\{\pi _d\}$ with $\pi _d(\varvec{\theta }) = \prod _{i=1}^d f(\theta _i)$ and $\varvec{\theta }' = \varvec{\theta }+ (\ell / \surd {d}) \varvec{\epsilon }$, *f* satisfying some regularity conditions. Under these assumptions, it can be proved that for *d* large,

3 $$\begin{aligned}&\mathbb {E}\left[ \min \left\{ 1, \frac{\pi _d(\varvec{\theta }')}{\pi _d(\varvec{\theta })}\right\} \right] \nonumber \\&\,\,\approx \mathbb {E}\left[ \min \left\{ 1, \exp \left( \sum _{i=1}^d \psi (\theta _i)(\theta _i' - \theta _i) - \frac{\ell ^2}{2d}\psi (\theta _i)^2\right) \right\} \right] \nonumber \\&\,\,= 2\mathbb {E}\left[ \varPhi \left( -\frac{\ell }{2}\sqrt{\frac{1}{d}\sum _{i=1}^d \psi (\theta _i)^2}\right) \right] , \end{aligned}$$

where “$\approx$” is to be understood as a relationship asserting that the expressions are asymptotically equivalent and

$$\begin{aligned} \psi (\theta _i) := \left. \frac{\partial }{\partial x} \log f(x)\right| _{x=\theta _i}; \end{aligned}$$

for the equality (3), we used that the term in the exponential has a conditional normal distribution given $\varvec{\theta }$ (because $\theta _i' - \theta _i = (\ell / \surd {d}) \epsilon _i$) and the closed-form of $\mathbb {E}[\min \{1, e^X\}]$ when $X \sim \varphi$. We establish a limit using that

$$\begin{aligned} 2\mathbb {E}\left[ \varPhi \left( -\frac{\ell }{2}\sqrt{\frac{1}{d}\sum _{i=1}^d \psi (\theta _i)^2}\right) \right] \rightarrow 2\varPhi (-\ell \surd {L}/2), \end{aligned}$$

with

$$\begin{aligned} L := \mathbb {E}[\psi (\theta _1)^2]. \end{aligned}$$

In their context, $\hat{\ell } = 2.38 / \surd {L}$ and $2 \, \varPhi \left( -\hat{\ell }\surd {L}/2\right) = 0.234$.

In our framework, we first consider a sequence of posterior densities $\{\pi _n\}$ based on observations of IID random variables $\mathbf {Y}_i \sim g_{\varvec{\theta }}$, $g_{\varvec{\theta }}$ satisfying some regularity conditions. Under Assumptions 1 and 2 and setting $\mathbf {S}_n = (\ell / \surd {d n}) \mathbf {M}_n$ with $\mathbf {M}_n\mathbf {M}_n^T = \mathcal {I}{(\hat{\varvec{\theta }}_n)}^{-1}$, it can be proved that for *n* large:

$$\begin{aligned}&\mathbb {E}\left[ \min \left\{ 1, \frac{\pi _n(\varvec{\theta }')}{\pi _n(\varvec{\theta })}\right\} \right] = \mathbb {E}\left[ \min \left\{ 1, \frac{\pi _n(\hat{\varvec{\theta }}_n + n^{-1/2}\mathbf {Z}'_n)}{\pi _n(\hat{\varvec{\theta }}_n + n^{-1/2}\mathbf {Z}_n)}\right\} \right] \\&\,\,\approx \mathbb {E}\left[ \min \left\{ 1, \frac{\pi _n(\varvec{\theta }_0 + n^{-1/2}\mathbf {Z}'_n)}{\pi _n(\varvec{\theta }_0 + n^{-1/2}\mathbf {Z}_n)}\right\} \right] \\&\,\,\approx \mathbb {E}\left[ \min \left\{ 1, \exp \left( -\frac{1}{2}\Vert \mathbf {Z}'_n\Vert _{\hat{\mathcal {I}}_n(\varvec{\theta }_0)}^2 + \frac{1}{2}\Vert \mathbf {Z}_n\Vert _{\hat{\mathcal {I}}_n(\varvec{\theta }_0)}^2\right) \right\} \right] , \end{aligned}$$

where

$$\begin{aligned} \hat{\mathcal {I}}_n(\varvec{\theta }_0) := \frac{1}{n}\sum _{i=1}^n-\left. \frac{\partial ^2}{\partial \varvec{\theta }\partial \varvec{\theta }^T} \log g_{\varvec{\theta }}(\mathbf {y}_i)\right| _{\varvec{\theta }= \varvec{\theta }_0}, \end{aligned}$$

using that $\hat{\varvec{\theta }}_n \rightarrow \varvec{\theta }_0$ and that the local asymptotic normality allows an expansion of $\log \pi _n(\varvec{\theta }_0 + n^{-1/2}\mathbf {z}_n)$ with vanishing terms beyond order 2. The last expectation above is asymptotically equivalent to

$$\begin{aligned} \mathbb {E}\left[ \min \left\{ 1, \frac{\varphi (\mathbf {Z}'; \mathbf {0}, \mathcal {I}{(\varvec{\theta }_0)}^{-1})}{\varphi (\mathbf {Z}; \mathbf {0}, \mathcal {I}{(\varvec{\theta }_0)}^{-1})}\right\} \right] , \end{aligned}$$

with $\mathbf {Z} \sim \varphi (\, \cdot \,; \mathbf {0}, \mathcal {I}{(\varvec{\theta }_0)}^{-1})$ and $\mathbf {Z}' \sim \varphi (\, \cdot \,; \mathbf {Z}, \lambda ^2 \mathbf {M} \mathbf {M}^T)$. The latter expectation is equal to (recall Corollary 1)

$$\begin{aligned} 2 \, \mathbb {E}\left[ \varPhi \left( - \frac{\ell \Vert \varvec{\epsilon }\Vert }{2 \surd {d}}\right) \right] \rightarrow 2 \, \varPhi \left( -\frac{\ell }{2}\right) , \end{aligned}$$

as $d \rightarrow \infty$. When $\mathbf {M}_n\mathbf {M}_n^T = \mathcal {I}{(\hat{\varvec{\theta }}_n)}^{-1}$, $L = 1$ because the proposal covariance is set to asymptotically match the target covariance exactly and thus $\hat{\ell } = 2.38$ with $2 \, \varPhi \left( -\hat{\ell }/2\right) = 0.234$. If, alternatively, the proposal is set to an isotropic Gaussian, i.e. $\mathbf {M}_n = \mathbf {1}$, a constant analogous to *L* appears in the limiting acceptance rate:

$$\begin{aligned} L' := \lim _{d\rightarrow \infty } \frac{\Vert \varvec{\epsilon }\Vert _{\mathcal {I}{(\varvec{\theta }_0)}}^2}{d}, \end{aligned}$$

provided that this limit exists (in distribution).

The formal results are presented in Proposition 2.

#### Proposition 2

(Guideline consistency) If $\varXi$ starts in stationarity, $\lambda = \ell /\surd {d}$ and $\mathbf {M} \mathbf {M}^T = \mathcal {I}{(\varvec{\theta }_0)}^{-1}$, then

$$\begin{aligned} \mathrm {ESJD}(\lambda , \mathbf {M})&:= \mathbb {E}\left[ \Vert \mathbf {Z}_{k + 1} - \mathbf {Z}_k\Vert _{\mathcal {I}{({\varvec{\theta }}_0)}}^2 \right] \\ {}&= 2\ell ^2 \, \mathbb {E}\left[ \frac{\Vert {\varvec{\epsilon }}\Vert ^2}{d} \varPhi \! \left( \!- \frac{\ell \Vert {\varvec{\epsilon }}\Vert }{2 \surd {d}}\right) \! \right] \! \rightarrow 2\ell ^2 \, \varPhi \left( \! - \frac{\ell }{2}\right) , \end{aligned}$$

and

$$\begin{aligned}&\mathbb {E}\left[ \min \left\{ 1, \frac{\varphi (\mathbf {Z}'; \mathbf {0}, \mathcal {I}{(\varvec{\theta }_0)}^{-1})}{\varphi (\mathbf {Z}; \mathbf {0}, \mathcal {I}{(\varvec{\theta }_0)}^{-1})}\right\} \right] \\&\quad = 2 \, \mathbb {E}\left[ \varPhi \left( - \frac{\ell \Vert \varvec{\epsilon }\Vert }{2 \surd {d}}\right) \right] \rightarrow 2 \, \varPhi \left( - \frac{\ell }{2}\right) , \end{aligned}$$

as $d \rightarrow \infty$, with $\mathbf {Z} \sim \varphi (\, \cdot \,; \mathbf {0}, \mathcal {I}{(\varvec{\theta }_0)}^{-1})$ and $\mathbf {Z}' \sim \varphi (\, \cdot \,; \mathbf {Z}, \lambda ^2 \mathbf {M} \mathbf {M}^T)$. Viewed as a function of $\ell$, $2\ell ^2 \, \varPhi \left( - \ell / 2\right)$ is maximized by $\ell = \hat{\ell } := 2.38$, and we obtain $2 \, \varPhi \left( -\hat{\ell }/2\right) = 0.234$.

In theory, one can obtain a more general limiting expression for $\mathrm {ESJD}(\lambda , \mathbf {M})$ when $\mathbf {M}$ is not specified to be such that $\mathbf {M} \mathbf {M}^T = \mathcal {I}{(\varvec{\theta }_0)}^{-1}$. However, one would need to know how $\mathcal {I}{(\varvec{\theta }_0)}^{-1}$ behaves when *d* grows because $\mathrm {ESJD}(\lambda , \mathbf {M})$ depends, in general, on $\mathcal {I}{(\varvec{\theta }_0)}^{-1}$. For example, from (1), it can be observed that

$$\begin{aligned} 2 \ell ^2 \, \mathbb {E}\left[ \frac{\Vert \varvec{\epsilon }\Vert _{\mathcal {I}{(\varvec{\theta }_0)}}^2}{d} \, \varPhi \left( -\frac{\ell \Vert \varvec{\epsilon }\Vert _{\mathcal {I}{(\varvec{\theta }_0)}}}{2 \surd {d}}\right) \right] \rightarrow 2\ell ^2 L' \, \varPhi \left( -\frac{\ell \surd L'}{2} \right) , \end{aligned}$$

whenever $\Vert \varvec{\epsilon }\Vert _{\mathcal {I}{(\varvec{\theta }_0)}}^2 / d \rightarrow L'\in \mathbb {R}$ as $d \rightarrow \infty$ in probability, that is, whenever the correlation in $\mathcal {I}{(\varvec{\theta }_0)}$ allows for a law of large numbers of the squared norm $\Vert \varvec{\epsilon }\Vert _{\mathcal {I}{(\varvec{\theta }_0)}}^2$, as long as uniform integrability conditions hold. In the previous section, for example, the autoregressive covariance matrix allows for a law of large numbers and uniform integrability conditions hold. This is a consequence of the form of $\mathcal {I}{(\varvec{\theta }_0)}$, which is a tridiagonal matrix, turning $\Vert \varvec{\epsilon }\Vert _{\mathcal {I}{(\varvec{\theta }_0)}}^2$ into a sum of correlated random variables, but where the correlation exists only for random variables that are close to each other; more precisely, each random variable in the sum is correlated with those with indices that differ by 1. The conditions aforementioned may fail to hold when the matrix $\mathcal {I}{(\varvec{\theta }_0)}$ yields a sum of correlated random variables where each of them is correlated to a number of random variables that grows with *d*.

The limiting behaviour of ESJD for the case $\mathbf {M} = \mathbf {1}$ recently received detailed attention in Yang et al. (2020). These authors perform analyses under the traditional asymptotic framework $d\rightarrow \infty$; however, in contrast to earlier work, their approach does not require the restrictive assumption of IID model parameters. Instead, the authors perform analyses under an assumption of partially connected graphical models. A key mathematical object there which measures the “roughness” of the log target density is

$$\begin{aligned} I_d(\theta ) := \frac{1}{d} \sum _{i=1}^d \left( \frac{\partial }{\partial \theta _i} \log \pi _d(\varvec{\theta })\right) ^2. \end{aligned}$$

It appears, for instance, in an expectation that is asymptotically equivalent to their expected acceptance probability:

4 $$\begin{aligned} 2 \mathbb {E}\left[ \varPhi \left( - \frac{\ell }{2} \sqrt{I_d(\varvec{\theta })}\right) \right] , \end{aligned}$$

where the expectation is with respect to $\pi _d$. It also appears in an expectation analogous to (1) that is asymptotically equivalent to their ESJD. There exists an interesting connection between their optimization problem and that of optimizing (1) that can be established by identifying the counterpart to $I_d(\theta )$ in (1) and the expected acceptance probability. The optimal acceptance rates derived under their framework are often close to 0.234, for large enough *d*, which is what we observed under our framework as well, for instance, in Sect. 3.2. We finish this section with a brief analysis which highlights the existence of that connection by focussing on similarities in between the acceptance rates.

We identify the counterpart to $I_d(\varvec{\theta })$ to be

$$\begin{aligned} \frac{\Vert \varvec{\epsilon }\Vert _{\mathcal {I}{(\varvec{\theta }_0)}}^2}{d}&= \frac{1}{d}\sum _{i=1}^d\sum _{j=1}^d \epsilon _i\epsilon _j \mathcal {I}{(\varvec{\theta }_0)}_{ij}, \end{aligned}$$

recalling that

$$\begin{aligned} \mathcal {I}{(\varvec{\theta })}_{ij} = \mathbb {E}\left[ \left( \frac{\partial }{\partial \theta _i}\log g_{\varvec{\theta }}(\mathbf {Y})\right) \left( \frac{\partial }{\partial \theta _j}\log g_{\varvec{\theta }}(\mathbf {Y})\right) \right] . \end{aligned}$$

Note that under regularity conditions, the normalized version of $\left( \frac{\partial }{\partial \theta _i} \log \pi _d(\varvec{\theta })\right) ^2$, when seen as the square of the derivative of the sum of the log prior and log densities, converges in distribution to $\mathcal {I}{(\varvec{\theta })}_{ii}$ times a chi-square random variable with 1 degree of freedom as $n \rightarrow \infty$. For weak interactions in between model parameters represented by sparse graphs, $\Vert \varvec{\epsilon }\Vert _{\mathcal {I}{(\varvec{\theta }_0)}}^2 / d$ thus encodes similar information to $I_d(\theta )$. This highlights that the expected acceptance probability under our framework, given by

$$\begin{aligned} 2 \, \mathbb {E}\left[ \varPhi \left( - \frac{\ell \Vert \varvec{\epsilon }\Vert _{\mathcal {I}{(\theta _0)}}}{2 \surd {d}}\right) \right] , \end{aligned}$$

and theirs, given by (4), are similar in essence. In general, Jensen’s inequality allows to observe that

$$\begin{aligned} 2 \, \mathbb {E}\left[ \varPhi \left( - \frac{\ell \Vert \varvec{\epsilon }\Vert _{\mathcal {I}{(\theta _0)}}}{2 \surd {d}}\right) \right] \ge 2 \, \varPhi \left( - \frac{\ell }{2} \sqrt{\frac{1}{d}\sum _{i=1}^d \mathcal {I}{(\varvec{\theta }_0)}_{ii}}\right) , \end{aligned}$$

given that $x \mapsto \varPhi (- a \sqrt{x})$ is convex for $x \ge 0$ with $a > 0$. Acceptance rates derived within our framework are thus expected to be larger than those derived within the framework of Yang et al. (2020), when $\pi _d$ concentrates around $\varvec{\theta }_0$. They have for instance been observed to be larger than 0.234 in Sect. 3.2, while in Yang et al. (2020) they are shown to be smaller than or equal to 0.234.

We do not investigate the problem of convergence of $\mathrm {ESJD}(\lambda , \mathbf {M})$ in full generality. In addition to Yang et al. (2020), we refer the reader to Ghosal (2000), Belloni and Chernozhukov (2009) and Belloni and Chernozhukov (2014) who conducted analyses of posterior distributions in asymptotic regimes where *d* is allowed to grow with *n*.

## Logistic regression with real data

In this section, we demonstrate that the RWM algorithm targeting $\pi _n$ behaves similarly to its asymptotic counterpart, targeting a Gaussian distribution, in some practical cases. To achieve this, we consider a specific practical case and compare the asymptotically optimal value for $\ell$ when $\mathbf {M} \mathbf {M}^T = \mathcal {I}{(\varvec{\theta }_0)}^{-1}$ based on ESJD (which does not depend on the unknown $\mathcal {I}{(\varvec{\theta }_0)}^{-1}$) to that obtained from tuning the non-limiting ESJD with $\mathbf {M}_n \mathbf {M}_n^T$ set to be the inverse of the observed information matrix. We also compare the optimal acceptance rates and present results for the RWM algorithm using $\mathbf {M}_n = \mathbf {1}$. The practical case that we study is one where the posterior distribution results from a Bayesian logistic regression model and a patent data set from Fahrmeir et al. (2007). We will see that for this example with a sample size of $n = 4{,}866$ and $d = 9$ parameters, both the optimal values for $\ell$ and acceptance rates coincide accurately, showing that the limiting RWM represents a good approximation of that targeting $\pi _n$ in situations where the Bayesian models are regular and the sample sizes are realistically large. This example also allows to show that the guidelines derived from the limiting RWM and the performance analysis conducted in Sect. 3.2 are relevant in such situations.

We denote the binary response variable and covariate vector data points by $r_1, \ldots , r_n$ and $\mathbf {x}_1, \ldots , \mathbf {x}_n$, respectively, with the first component of each $\mathbf {x}_i$ being equal to 1. In logistic regression, the parameters $\varvec{\theta }$ are regression coefficients. Let us assume that $\mathbf {Y}_1, \ldots , \mathbf {Y}_n = (R_1, \mathbf {X}_1), \ldots , (R_n, \mathbf {X}_n)$ are IID random variables and also that the model is well specified in order to fit in the theoretical framework presented in Sect. 2. Formally speaking, the latter assumption is certainly not true, but the fact that the empirical results are close to the theoretical (and asymptotic) ones suggests that the model approximates well the true data generating process. We now show that Theorem 1 can be applied by verifying the assumptions stated in Sect. 2. The logistic regression model is, as mentioned in Sect. 1.3, regular enough; Assumption 1 is thus satisfied. We set $\mathbf {M}_n \mathbf {M}_n^T$ to be the inverse of a standardized version of the observed information matrix evaluated at the maximum a posteriori estimate $\hat{\varvec{\theta }}_n$, i.e. the inverse of5Under weak regularity conditions, $\mathbf {M}_n \mathbf {M}_n^T$ converges and we set $\mathbf {S}_n = (\lambda / \surd {n}) \mathbf {M}_n$, implying that Assumption 2 is satisfied if these weak regularity conditions are verified. Theorem 1 therefore holds provided that the chains start in stationarity (Assumption 3) and these weak regularity conditions are verified.

When $d = 9$, the asymptotically optimal value for $\ell$ when $\mathbf {M} \mathbf {M}^T = \mathcal {I}{(\varvec{\theta }_0)}^{-1}$ is 2.39 and the acceptance rate of the limiting RWM using this value is $26.26\%$. The optimal values for the RWM algorithm with $\mathbf {M}_n$ set as the inverse of (5) are essentially the same: 2.37 and $26.68\%$ for $\ell$ and the acceptance rate, respectively. The value of $\ell$ that maximizes the ESS per iteration is 2.40; the maximum ESS per iteration is 0.034, which is significantly higher than the maximum of 0.006 attained by the algorithm with $\mathbf {M}_n = \mathbf {1}$. As explained and shown in Sect. 3, a poor performance of the latter sampler is due to a strong correlation in between the parameters. For this sampler, a value of 6.89 is optimal for $\ell$ based on the ESS, whereas a value of 6.51 is optimal when the ESJD is instead considered. The acceptance rate of the algorithm using $\mathbf {M}_n = \mathbf {1}$ and the latter value is $27.69\%$. Note that we tried smaller models with less covariates and larger ones with interaction terms, and the optimal values when $\mathbf {M}_n$ is set as the inverse of (5) are consistent with the guidelines presented in Table 1. The results in this numerical experiment follow from a numerical optimization of ESJD and ESS based on Markov chain samples of size 10,000,000 and a grid search.

## Discussion

In this paper, we have analysed the behaviour of random walk Metropolis (RWM) algorithms when used to sample from Bayesian posterior distributions, under the asymptotic regime $n \rightarrow \infty$, in contrast with previous asymptotic analyses where $d\rightarrow \infty$. Our analysis led to novel parameter-dimension-dependent tuning guidelines which are consistent with the well-known 0.234 rule. A formal argument allowed to show that this rule can in fact be derived from the angle adopted in this paper as well. We believe that similar analyses to those performed in this paper can be conducted to develop practical tuning guidelines for more sophisticated algorithms like Metropolis-adjusted Langevin algorithm (Roberts and Tweedie 1996) and Hamiltonian Monte Carlo (Duane et al. 1987), and to establish other interesting connections with optimal scaling literature (e.g. Roberts and Rosenthal 1998; Beskos et al. 2013).

The guidelines developed in this paper for RWM algorithms are valid under weak assumptions; we essentially only require a Bernstein–von Mises theorem to hold for the target distribution. This is in stark contrast to scaling limit approaches. To our knowledge, there is one contribution, Yang et al. (2020), that provides guidelines for a realistic model based on a scaling limit argument, and it requires the posterior distribution to concentrate, which is in line with the argument of this paper. The guidelines proposed in our paper are in theory valid in the limit $n \rightarrow \infty$; we have demonstrated that they are nevertheless applicable in realistic scenarios with typical data sizes using an example of logistic-regression analysis of real data. This example, together with our analysis of the limiting RWM, also allows to support the findings about the robustness of the 0.234 rule to non-independent and identically distributed (IID) model parameters when the scaling matrix is a diagonal matrix.

## A Proofs

### Proof (Theorem 1)

*Result (i).* To prove this result, we use Theorem 2 of Schmon et al. (2021a). We thus have to verify three conditions.

1. As $n \rightarrow \infty$, the following convergence holds in $\mathbb {P}^\mathbf {Y}$-probability: $\mathbf {Z}_{0, n}$ converges weakly to $\mathbf {Z}_{0}$.
2. Use $P_n$ and *P* to denote the transition kernels of $\varXi _{n}$ and $\varXi$, respectively. These are such that
  $$\begin{aligned} \int |P_nh(\mathbf {z}) - Ph(\mathbf {z})| \, \pi _{\mathbf {Z}_n}(\mathbf {z}) \, \mathrm {d}\mathbf {z} \rightarrow 0, \end{aligned}$$
  in $\mathbb {P}^\mathbf {Y}$-probability as $n \rightarrow \infty$, for all $h \in \text {BL}$, the set of bounded Lipschitz functions.
3. The transition kernel *P* is such that $Ph(\, \cdot \,)$ is continuous for any $h \in \mathcal {C}_{\text {b}}$, the set of continuous bounded functions.

We start with Condition 1. It suffices to verify that

$$\begin{aligned} |\mathbb {P}(\mathbf {Z}_{0, n} \in A) - \mathbb {P}(\mathbf {Z}_{0} \in A)| \rightarrow 0, \end{aligned}$$

in $\mathbb {P}^\mathbf {Y}$-probability, for any measurable set *A*. We have that

$$\begin{aligned}&|\mathbb {P}(\mathbf {Z}_{0, n} \in A) - \mathbb {P}(\mathbf {Z}_{0} \in A)| \le \int \left| {\pi }_{n}(\varvec{\theta })\right. \\&\left. -\varphi (\varvec{\theta }; \hat{\varvec{\theta }}_{n}, \mathcal {I}{(\varvec{\theta }_0)}^{-1}/n)\right| \mathrm {d}\varvec{\theta }\rightarrow 0 \end{aligned}$$

in $\mathbb {P}^\mathbf {Y}$-probability by Assumption 1, using Jensen’s inequality, that $A \subseteq {{\,\mathrm{\mathbb {R}}\,}}^d$, and a change of variable $\varvec{\theta }= \mathbf {z} / n^{1/2} + \hat{\varvec{\theta }}_n$.

We turn to Condition 2. We have that

$$\begin{aligned} P_n(\mathbf {z}, \mathrm {d}\mathbf {z}') = \alpha _n(\mathbf {z}, \mathbf {z}') \, \varphi (\mathrm {d}\mathbf {z}'; \mathbf {z}, \lambda ^2 \mathbf {M}_n \mathbf {M}_n^T) + \rho _n(\mathbf {z}) \, \delta _{\mathbf {z}}(\mathrm {d}\mathbf {z}'), \end{aligned}$$

and

$$\begin{aligned} P(\mathbf {z}, \mathrm {d}\mathbf {z}') = \alpha (\mathbf {z}, \mathbf {z}') \, \varphi (\mathrm {d}\mathbf {z}'; \mathbf {z}, \lambda ^2 \mathbf {M} \mathbf {M}^T) + \rho (\mathbf {z}) \, \delta _{\mathbf {z}}(\mathrm {d}\mathbf {z}'), \end{aligned}$$

where here

$$\begin{aligned} \alpha _n(\mathbf {z}, \mathbf {z}') := \min \left\{ 1, \frac{\pi _{\mathbf {Z}_n}(\mathbf {z}')}{\pi _{\mathbf {Z}_n}(\mathbf {z})}\right\} , \end{aligned}$$

$\rho _n(\mathbf {z})$ is the corresponding rejection probability, and

$$\begin{aligned} \alpha (\mathbf {z}, \mathbf {z}') := \min \left\{ 1, \frac{\varphi (\mathbf {z}'; \mathbf {0}, \mathcal {I}{(\varvec{\theta }_0)}^{-1})}{\varphi (\mathbf {z}; \mathbf {0}, \mathcal {I}{(\varvec{\theta }_0)}^{-1})}\right\} , \end{aligned}$$

$\rho (\mathbf {z})$ is the corresponding rejection probability. Thus,

$$\begin{aligned} P_nh(\mathbf {z}) =&\int h(\mathbf {z}') \, \alpha _n(\mathbf {z}, \mathbf {z}') \, \varphi (\mathrm {d}\mathbf {z}'; \mathbf {z}, \lambda ^2 \mathbf {M}_n \mathbf {M}_n^T)\\&+ h(\mathbf {z}) \, \rho _n(\mathbf {z}), \end{aligned}$$

and

$$\begin{aligned} Ph(\mathbf {z}) = \int h(\mathbf {z}') \, \alpha (\mathbf {z}, \mathbf {z}') \, \varphi (\mathrm {d}\mathbf {z}'; \mathbf {z}, \lambda ^2 \mathbf {M} \mathbf {M}^T) + h(\mathbf {z}) \, \rho (\mathbf {z}). \end{aligned}$$

Therefore, using the triangle inequality,

$$\begin{aligned}&\int |P_nh(\mathbf {z}) - Ph(\mathbf {z})| \, \pi _{\mathbf {Z}_n}(\mathbf {z}) \, \mathrm {d}\mathbf {z} \\&\quad \le \int \left| \int h(\mathbf {z}') \, \alpha _n(\mathbf {z}, \mathbf {z}') \, \varphi (\mathrm {d}\mathbf {z}'; \mathbf {z}, \lambda ^2 \mathbf {M}_n \mathbf {M}_n^T)\right. \\&\qquad \left. - \int h(\mathbf {z}') \, \alpha (\mathbf {z}, \mathbf {z}') \,\right. \\&\left. \varphi (\mathrm {d}\mathbf {z}'; \mathbf {z}, \lambda ^2 \mathbf {M} \mathbf {M}^T)\right| \, \pi _{\mathbf {Z}_n}(\mathbf {z}) \, \mathrm {d}\mathbf {z} \\&\qquad +\, \int |h(\mathbf {z}) \, \rho _n(\mathbf {z}) - h(\mathbf {z}) \, \rho (\mathbf {z})| \, \pi _{\mathbf {Z}_n}(\mathbf {z}) \, \mathrm {d}\mathbf {z}. \end{aligned}$$

We prove that the first integral on the right-hand side (RHS) converges to 0 in $\mathbb {P}^\mathbf {Y}$-probability. The other integral is seen to converge using similar arguments.

We have that

6 $$\begin{aligned}&\int \left| \int h(\mathbf {z}') \, \alpha _n(\mathbf {z}, \mathbf {z}') \, \varphi (\mathrm {d}\mathbf {z}'; \mathbf {z}, \lambda ^2 \mathbf {M}_n \mathbf {M}_n^T) \nonumber \right. \\&\left. - \int h(\mathbf {z}') \, \alpha (\mathbf {z}, \mathbf {z}') \, \varphi (\mathrm {d}\mathbf {z}'; \mathbf {z}, \lambda ^2 \mathbf {M} \mathbf {M}^T)\right| \,\nonumber \\&\pi _{\mathbf {Z}_n}(\mathbf {z}) \, \mathrm {d}\mathbf {z} \nonumber \\&\quad \le K \iint \left| \alpha _n(\mathbf {z}, \mathbf {z}') \, \varphi (\mathrm {d}\mathbf {z}'; \mathbf {z}, \lambda ^2 \mathbf {M}_n \mathbf {M}_n^T) \nonumber \right. \\&\left. - \alpha (\mathbf {z}, \mathbf {z}') \, \varphi (\mathrm {d}\mathbf {z}'; \mathbf {z}, \lambda ^2 \mathbf {M} \mathbf {M}^T)\right| \,\nonumber \\&\pi _{\mathbf {Z}_n}(\mathbf {z}) \, \mathrm {d}\mathbf {z} \nonumber \\&\quad \le K \iint \left| \alpha _n(\mathbf {z}, \mathbf {z}') \, \varphi (\mathrm {d}\mathbf {z}'; \mathbf {z}, \lambda ^2 \mathbf {M}_n \mathbf {M}_n^T) - \alpha _n(\mathbf {z}, \mathbf {z}') \,\nonumber \right. \\&\left. \varphi (\mathrm {d}\mathbf {z}'; \mathbf {z}, \lambda ^2 \mathbf {M} \mathbf {M}^T)\right| \,\nonumber \\&\pi _{\mathbf {Z}_n}(\mathbf {z}) \, \mathrm {d}\mathbf {z} \nonumber \\&\qquad + K \iint \left| \alpha _n(\mathbf {z}, \mathbf {z}') \, \varphi (\mathrm {d}\mathbf {z}'; \mathbf {z}, \lambda ^2 \mathbf {M} \mathbf {M}^T)\nonumber \right. \\&\left. - \alpha (\mathbf {z}, \mathbf {z}') \, \varphi (\mathrm {d}\mathbf {z}'; \mathbf {z}, \lambda ^2 \mathbf {M} \mathbf {M}^T)\right| \, \pi _{\mathbf {Z}_n}(\mathbf {z}) \, \mathrm {d}\mathbf {z}, \end{aligned}$$

using Jensen’s inequality, that there exists a positive constant *K* such that $|h| \le K$, and the triangle inequality. We now prove that each of the last two integrals converges to 0. We begin with the first one:

$$\begin{aligned}&\iint \left| \alpha _n(\mathbf {z}, \mathbf {z}') \, \varphi (\mathrm {d}\mathbf {z}'; \mathbf {z}, \lambda ^2 \mathbf {M}_n \mathbf {M}_n^T) - \alpha _n(\mathbf {z}, \mathbf {z}') \,\right. \\&\left. \varphi (\mathrm {d}\mathbf {z}'; \mathbf {z}, \lambda ^2 \mathbf {M} \mathbf {M}^T)\right| \, \pi _{\mathbf {Z}_n}(\mathbf {z}) \, \mathrm {d}\mathbf {z} \\&\quad \le \iint \left| \varphi (\mathrm {d}\mathbf {z}'; \mathbf {z}, \lambda ^2 \mathbf {M}_n \mathbf {M}_n^T) \right. \\&\qquad \left. - \varphi (\mathrm {d}\mathbf {z}'; \mathbf {z}, \lambda ^2 \mathbf {M} \mathbf {M}^T)\right| \, \pi _{\mathbf {Z}_n}(\mathbf {z}) \, \mathrm {d}\mathbf {z} \\&\quad \le \left[ \text {tr}((\mathbf {M} \mathbf {M}^T)^{-1}\mathbf {M}_n \mathbf {M}_n^T - \mathbf {1})\right. \\&\qquad \left. - \log \det (\mathbf {M}_n \mathbf {M}_n^T (\mathbf {M} \mathbf {M}^T)^{-1}) \right] ^{1/2} \rightarrow 0, \end{aligned}$$

in $\mathbb {P}^\mathbf {Y}$-probability by Assumption 2, using that $0 \le \alpha _n \le 1$ and Devroye et al., (2018, Proposition 2.1), where $\text {tr}(\, \cdot \,)$ and $\det (\, \cdot \,)$ are the trace and determinant operators, respectively. Note that by Assumption 2 we have that $\mathbf {M}_n \mathbf {M}_n^T \rightarrow \mathbf {M} \mathbf {M}^T$ in probability, meaning that all components converge, which implies that the trace and the log of the determinant both vanish.

Next,

$$\begin{aligned}&\iint \left| \alpha _n(\mathbf {z}, \mathbf {z}') \, \varphi (\mathrm {d}\mathbf {z}'; \mathbf {z}, \lambda ^2 \mathbf {M} \mathbf {M}^T) \right. \\&\qquad \left. - \alpha (\mathbf {z}, \mathbf {z}') \, \varphi (\mathrm {d}\mathbf {z}'; \mathbf {z}, \lambda ^2 \mathbf {M} \mathbf {M}^T)\right| \, \pi _{\mathbf {Z}_n}(\mathbf {z}) \, \mathrm {d}\mathbf {z} \\&\quad \le \iint \left| \alpha _n(\mathbf {z}, \mathbf {z}') \, \pi _{\mathbf {Z}_n}(\mathbf {z}) \right. \\&\qquad \left. - \alpha (\mathbf {z}, \mathbf {z}') \, \varphi (\mathbf {z}; \mathbf {0}, \mathcal {I}{(\varvec{\theta }_0)}^{-1})\right| \, \varphi (\mathrm {d}\mathbf {z}'; \mathbf {z}, \lambda ^2 \mathbf {M} \mathbf {M}^T) \, \mathrm {d}\mathbf {z} \\&\qquad + \iint \left| \alpha (\mathbf {z}, \mathbf {z}') \, \varphi (\mathbf {z}; \mathbf {0}, \mathcal {I}{(\varvec{\theta }_0)}^{-1}) \right. \\&\qquad \left. - \alpha (\mathbf {z}, \mathbf {z}') \, \pi _{\mathbf {Z}_n}(\mathbf {z})\right| \, \varphi (\mathrm {d}\mathbf {z}'; \mathbf {z}, \lambda ^2 \mathbf {M} \mathbf {M}^T) \, \mathrm {d}\mathbf {z}, \end{aligned}$$

using the triangle inequality. The second integral is seen to converge to 0 because

7 $$\begin{aligned} &\iint \left| \alpha (\mathbf {z}, \mathbf {z}') \, \varphi (\mathbf {z}; \mathbf {0}, \nonumber \right. \\&\qquad \left. \mathcal {I}{(\varvec{\theta }_0)}^{-1}) - \alpha (\mathbf {z}, \mathbf {z}') \, \pi _{\mathbf {Z}_n}(\mathbf {z})\right| \, \varphi (\mathrm {d}\mathbf {z}'; \mathbf {z}, \lambda ^2 \mathbf {M} \mathbf {M}^T) \, \mathrm {d}\mathbf {z} \nonumber \\&\quad \le \int \left| \varphi (\mathbf {z}; \mathbf {0}, \mathcal {I}{(\varvec{\theta }_0)}^{-1}) - \pi _{\mathbf {Z}_n}(\mathbf {z})\right| \, \mathrm {d}\mathbf {z} \nonumber \\&\quad = \int \left| \varphi (\varvec{\theta }; \hat{\varvec{\theta }}_{n}, \mathcal {I}{(\varvec{\theta }_0)}^{-1}/n) - {\pi }_{n}(\varvec{\theta })\right| \mathrm {d}\varvec{\theta }\rightarrow 0 \end{aligned}$$

in $\mathbb {P}^\mathbf {Y}$-probability by Assumption 1, using that $0 \le \alpha \le 1$ and a change of variable $\varvec{\theta }= \mathbf {z} / n^{1/2} + \hat{\varvec{\theta }}_n$. For the first integral, we write

$$\begin{aligned}&\iint \left| \alpha _n(\mathbf {z}, \mathbf {z}') \, \pi _{\mathbf {Z}_n}(\mathbf {z}) - \alpha (\mathbf {z}, \mathbf {z}') \, \varphi (\mathbf {z}; \mathbf {0}, \mathcal {I}{(\varvec{\theta }_0)}^{-1})\right| \\&\, \varphi (\mathrm {d}\mathbf {z}'; \mathbf {z}, \lambda ^2 \mathbf {M} \mathbf {M}^T) \, \mathrm {d}\mathbf {z} \\&\quad = \iint \left| \min \{\pi _{\mathbf {Z}_n}(\mathbf {z}), \pi _{\mathbf {Z}_n}(\mathbf {z}')\} \right. \\&\qquad \left. - \min \{\varphi (\mathbf {z}; \mathbf {0},\mathcal {I}{(\varvec{\theta }_0)}^{-1}), \varphi (\mathbf {z}'; \mathbf {0}, \mathcal {I}{(\varvec{\theta }_0)}^{-1})\}\right| \\&\, \varphi (\mathrm {d}\mathbf {z}'; \mathbf {z}, \lambda ^2 \mathbf {M} \mathbf {M}^T) \, \mathrm {d}\mathbf {z} \\&\quad \le \iint \left| \pi _{\mathbf {Z}_n}(\mathbf {z}) - \varphi (\mathbf {z}; \mathbf {0},\mathcal {I}{(\varvec{\theta }_0)}^{-1})\right| \, \varphi (\mathrm {d}\mathbf {z}'; \mathbf {z}, \lambda ^2 \mathbf {M} \mathbf {M}^T) \, \mathrm {d}\mathbf {z} \\&\qquad + \iint \left| \pi _{\mathbf {Z}_n}(\mathbf {z}') - \varphi (\mathbf {z}'; \mathbf {0}, \mathcal {I}{(\varvec{\theta }_0)}^{-1})\right| \,\\&\varphi (\mathrm {d}\mathbf {z}'; \mathbf {z}, \lambda ^2 \mathbf {M} \mathbf {M}^T) \, \mathrm {d}\mathbf {z}, \end{aligned}$$

using that $|\min \{a, b\} - \min \{c, d\}| \le |a - c| + |b - d|$ for any real numbers *a*, *b*, *c* and *d*. It is seen that both integrals on the RHS vanish as above (recall (7)) after noticing that $\varphi (\mathrm {d}\mathbf {z}'; \mathbf {z}, \lambda ^2 \mathbf {M} \mathbf {M}^T) \, \mathrm {d}\mathbf {z} = \varphi (\mathrm {d}\mathbf {z}; \mathbf {z}', \lambda ^2 \mathbf {M} \mathbf {M}^T) \, \mathrm {d}\mathbf {z}'$, which is used in the second integral.

There remains to verify Condition 3: the continuity of *Ph*. Without loss of generality, consider a non-random sequence of vectors $(\mathbf {e}_n)_{n \ge 1}$ with monotonically shrinking components (in absolute value) such that $\sup _n \mathbf {e}_n^T (\mathbf {M} \mathbf {M}^T)^{-1} \mathbf {e}_n < \infty$. We now prove that $Ph(\mathbf {z}+ \mathbf {e}_n) \rightarrow Ph(\mathbf {z})$ as $n \rightarrow \infty$.

We have that

$$\begin{aligned}&Ph(\mathbf {z} + \mathbf {e}_n) = \int h(\mathbf {z}') \, \alpha (\mathbf {z}+ \mathbf {e}_n, \mathbf {z}') \, \varphi (\mathrm {d}\mathbf {z}'; \mathbf {z}\\&+ \mathbf {e}_n, \lambda ^2 \mathbf {M} \mathbf {M}^T) + h(\mathbf {z}+ \mathbf {e}_n) \, \rho (\mathbf {z}+ \mathbf {e}_n). \end{aligned}$$

We prove that the first term on the RHS converges to

$$\begin{aligned} \int h(\mathbf {z}') \, \alpha (\mathbf {z}, \mathbf {z}') \, \varphi (\mathrm {d}\mathbf {z}'; \mathbf {z}, \lambda ^2 \mathbf {M} \mathbf {M}^T); \end{aligned}$$

the convergence of the second term follows using similar arguments.

We write

$$\begin{aligned}&\int h(\mathbf {z}') \, \alpha (\mathbf {z}+ \mathbf {e}_n, \mathbf {z}') \, \varphi (\mathrm {d}\mathbf {z}'; \mathbf {z}+ \mathbf {e}_n, \lambda ^2 \mathbf {M} \mathbf {M}^T) \\&\quad = \exp \left\{ -\frac{\mathbf {e}_n^T (\lambda ^2 \mathbf {M} \mathbf {M}^T)^{-1} \mathbf {e}_n}{2}\right\} \\&\int h(\mathbf {z}') \, \alpha (\mathbf {z}+ \mathbf {e}_n, \mathbf {z}') \, \exp \left\{ -\mathbf {e}_n^T (\lambda ^2 \mathbf {M} \mathbf {M}^T)^{-1} (\mathbf {z}' - \mathbf {z})\right\} \,\\&\varphi (\mathrm {d}\mathbf {z}'; \mathbf {z}, \lambda ^2 \mathbf {M} \mathbf {M}^T) \\&\quad =\exp \left\{ -\frac{\mathbf {e}_n^T (\lambda ^2 \mathbf {M} \mathbf {M}^T)^{-1} \mathbf {e}_n}{2}\right\} \\&\mathbb {E}_{\mathbf {z}}\left[ h(\mathbf {Z}') \, \alpha (\mathbf {z}+ \mathbf {e}_n, \mathbf {Z}') \, \right. \\&\left. \exp \left\{ -\mathbf {e}_n^T (\lambda ^2 \mathbf {M} \mathbf {M}^T)^{-1} (\mathbf {Z}' - \mathbf {z})\right\} \right] , \end{aligned}$$

where the expectation is with respect to $\varphi (\, \cdot \,; \mathbf {z}, \lambda ^2 \mathbf {M} \mathbf {M}^T)$; we highlight a dependence on $\mathbf {z}$ using the notation $\mathbb {E}_{\mathbf {z}}$.

We have that

$$\begin{aligned} \exp \left\{ -\frac{\mathbf {e}_n^T (\lambda ^2 \mathbf {M} \mathbf {M}^T)^{-1} \mathbf {e}_n}{2}\right\} \rightarrow 1, \end{aligned}$$

and

$$\begin{aligned}&h(\mathbf {Z}') \, \alpha (\mathbf {z}+ \mathbf {e}_n, \mathbf {Z}') \, \exp \left\{ -\mathbf {e}_n^T (\lambda ^2 \mathbf {M} \mathbf {M}^T)^{-1} (\mathbf {Z}' - \mathbf {z})\right\} \\&\qquad \rightarrow h(\mathbf {Z}') \, \alpha (\mathbf {z}, \mathbf {Z}'), \end{aligned}$$

almost surely, given the continuity of $\alpha$ and the exponential function.

To prove that the expectation converges to

$$\begin{aligned} \mathbb {E}_{\mathbf {z}}\left[ h(\mathbf {Z}') \, \alpha (\mathbf {z}, \mathbf {Z}')\right] = \int h(\mathbf {z}') \, \alpha (\mathbf {z}, \mathbf {z}') \, \varphi (\mathrm {d}\mathbf {z}'; \mathbf {z}, \lambda ^2 \mathbf {M} \mathbf {M}^T), \end{aligned}$$

we thus only need to prove that

$$\begin{aligned} h(\mathbf {Z}') \, \alpha (\mathbf {z}+ \mathbf {e}_n, \mathbf {Z}') \, \exp \left\{ -\mathbf {e}_n^T (\lambda ^2 \mathbf {M} \mathbf {M}^T)^{-1} (\mathbf {Z}' - \mathbf {z})\right\} \end{aligned}$$

is uniformly integrable. To prove this, we show that

$$\begin{aligned}&\sup _n \mathbb {E}\left[ \left( h(\mathbf {Z}') \, \alpha (\mathbf {z}+ \mathbf {e}_n, \mathbf {Z}') \, \exp \left\{ -\mathbf {e}_n^T (\lambda ^2 \mathbf {M} \mathbf {M}^T)^{-1} (\mathbf {Z}' - \mathbf {z})\right\} \right) ^2\right] \\&\qquad < \infty . \end{aligned}$$

We have that

$$\begin{aligned}&\mathbb {E}\left[ \left( h(\mathbf {Z}') \, \alpha (\mathbf {z}+ \mathbf {e}_n, \mathbf {Z}') \, \exp \left\{ -\mathbf {e}_n^T (\lambda ^2 \mathbf {M} \mathbf {M}^T)^{-1} (\mathbf {Z}' - \mathbf {z})\right\} \right) ^2\right] \\&\qquad \le K^2 \mathbb {E}\left[ \exp \left\{ -2\,\mathbf {e}_n^T (\lambda ^2 \mathbf {M} \mathbf {M}^T)^{-1} (\mathbf {Z}' - \mathbf {z})\right\} \right] \\&= K^2 \exp \left\{ 2 \, \lambda ^{-2} \, \mathbf {e}_n^T (\mathbf {M} \mathbf {M}^T)^{-1} \mathbf {e}_n\right\} . \end{aligned}$$

This concludes the proof of Result (i).

*Result (ii).* We want to prove that

$$\begin{aligned}&\left| \iint \alpha _n(\mathbf {z}, \mathbf {z}') \, \varphi (\mathrm {d}\mathbf {z}'; \mathbf {z}, \lambda ^2 \mathbf {M}_n \mathbf {M}_n^T) \, \pi _{\mathbf {Z}_n}(\mathbf {z}) \, \mathrm {d}\mathbf {z}\right. \\&\quad \left. - \iint \alpha (\mathbf {z}, \mathbf {z}') \, \varphi (\mathrm {d}\mathbf {z}'; \mathbf {z}, \lambda ^2 \mathbf {M} \mathbf {M}^T) \, \varphi (\mathrm {d}\mathbf {z}; \mathbf {0}, \mathcal {I}{(\varvec{\theta }_0)}^{-1})\right| \\&\quad \rightarrow 0, \end{aligned}$$

in $\mathbb {P}^\mathbf {Y}$-probability as $n \rightarrow \infty$. Using the triangle and Jensen’s inequality and that $0 \le \alpha \le 1$,

$$\begin{aligned}&\left| \iint \alpha _n(\mathbf {z}, \mathbf {z}') \, \varphi (\mathrm {d}\mathbf {z}'; \mathbf {z}, \lambda ^2 \mathbf {M}_n \mathbf {M}_n^T) \, \pi _{\mathbf {Z}_n}(\mathbf {z}) \, \mathrm {d}\mathbf {z}\right. \\&\qquad \left. - \iint \alpha (\mathbf {z}, \mathbf {z}') \, \varphi (\mathrm {d}\mathbf {z}'; \mathbf {z}, \lambda ^2 \mathbf {M} \mathbf {M}^T) \, \varphi (\mathrm {d}\mathbf {z}; \mathbf {0}, \mathcal {I}{(\varvec{\theta }_0)}^{-1})\right| \\&\quad \le \left| \iint \alpha _n(\mathbf {z}, \mathbf {z}') \, \varphi (\mathrm {d}\mathbf {z}'; \mathbf {z}, \lambda ^2 \mathbf {M}_n \mathbf {M}_n^T) \, \pi _{\mathbf {Z}_n}(\mathbf {z}) \, \mathrm {d}\mathbf {z} \right. \\&\qquad \left. - \iint \alpha (\mathbf {z}, \mathbf {z}') \, \varphi (\mathrm {d}\mathbf {z}'; \mathbf {z}, \lambda ^2 \mathbf {M} \mathbf {M}^T) \, \pi _{\mathbf {Z}_n}(\mathbf {z}) \, \mathrm {d}\mathbf {z}\right| \\&\qquad + \left| \iint \alpha (\mathbf {z}, \mathbf {z}') \, \varphi (\mathrm {d}\mathbf {z}'; \mathbf {z}, \lambda ^2 \mathbf {M} \mathbf {M}^T) \, \pi _{\mathbf {Z}_n}(\mathbf {z}) \, \mathrm {d}\mathbf {z}\right. \\&\qquad \left. - \iint \alpha (\mathbf {z}, \mathbf {z}') \, \varphi (\mathrm {d}\mathbf {z}'; \mathbf {z},\right. \\&\left. \lambda ^2 \mathbf {M} \mathbf {M}^T) \, \varphi (\mathrm {d}\mathbf {z}; \mathbf {0}, \mathcal {I}{(\varvec{\theta }_0)}^{-1})\right| \\&\quad \le \iint \left| \alpha _n(\mathbf {z}, \mathbf {z}') \, \varphi (\mathrm {d}\mathbf {z}'; \mathbf {z}, \lambda ^2 \mathbf {M}_n \mathbf {M}_n^T) \right. \\&\qquad \left. - \alpha (\mathbf {z}, \mathbf {z}') \, \varphi (\mathrm {d}\mathbf {z}'; \mathbf {z}, \lambda ^2 \mathbf {M} \mathbf {M}^T) \right| \, \pi _{\mathbf {Z}_n}(\mathbf {z}) \, \mathrm {d}\mathbf {z} \\&\qquad + \int \left| \pi _{\mathbf {Z}_n}(\mathbf {z}) - \varphi (\mathbf {z}; \mathbf {0}, \mathcal {I}{(\varvec{\theta }_0)}^{-1})\right| \, \mathrm {d}\mathbf {z}. \end{aligned}$$

We have shown in the proof of Result (i) that both integrals converge to 0 (recall (6) and (7)), which concludes the proof of Result (ii).

*Result (iii).* To prove this result, we show that

$$\begin{aligned}&\mathbb {E}\left[ \Vert \lambda \mathbf {M}_n \varvec{\epsilon }\Vert _{\mathcal {I}{(\hat{\varvec{\theta }}_n)}}^2 \alpha _n(\mathbf {Z}_n, \mathbf {Z}_n + \lambda \mathbf {M}_n \varvec{\epsilon })\right] \\&- \mathbb {E}\left[ \Vert \lambda \mathbf {M} \varvec{\epsilon }\Vert _{\mathcal {I}{(\varvec{\theta }_0)}}^2 \alpha (\mathbf {Z}, \mathbf {Z} + \lambda \mathbf {M} \varvec{\epsilon })\right] \rightarrow 0, \end{aligned}$$

in $\mathbb {P}^\mathbf {Y}$-probability, where $\mathbf {Z}_n \sim \pi _{\mathbf {Z}_n}$ and $\mathbf {Z} \sim \varphi (\, \cdot \,; \mathbf {0}, \mathcal {I}{(\varvec{\theta }_0)}^{-1})$, under the assumption that

$$\begin{aligned}&\left| \mathbb {E}\left[ \Vert \lambda \mathbf {M}_n \varvec{\epsilon }\Vert _{\mathcal {I}{(\hat{\varvec{\theta }}_n)}}^2 \alpha _n(\mathbf {Z}_n, \mathbf {Z}_n + \lambda \mathbf {M}_n \varvec{\epsilon })\right] \right. \\&\left. - \mathbb {E}\left[ \Vert \lambda \mathbf {M}\varvec{\epsilon }\Vert _{\mathcal {I}{(\varvec{\theta }_0)}}^2 \alpha _n(\mathbf {Z}_n, \mathbf {Z}_n + \lambda \mathbf {M}\varvec{\epsilon })\right] \right| \rightarrow 0, \end{aligned}$$

which will be seen to imply Result (iii). Indeed, this assumption is more general than $\mathbf {M}_n \mathbf {M}_n^T = \mathcal {I}{(\hat{\varvec{\theta }}_n)}^{-1} \rightarrow \mathbf {M}\mathbf {M}^T = \mathcal {I}{(\varvec{\theta }_0)}^{-1}$; we will show below that it is verified when $\mathbf {M}_n \mathbf {M}_n^T = \mathcal {I}{(\hat{\varvec{\theta }}_n)}^{-1} \rightarrow \mathbf {M}\mathbf {M}^T = \mathcal {I}{(\varvec{\theta }_0)}^{-1}$.

Using the triangle inequality,

$$\begin{aligned}&\left| \mathbb {E}\left[ \Vert \lambda \mathbf {M}_n \varvec{\epsilon }\Vert _{\mathcal {I}{(\hat{\varvec{\theta }}_n)}}^2 \alpha _n(\mathbf {Z}_n, \mathbf {Z}_n + \lambda \mathbf {M}_n \varvec{\epsilon })\right] \right. \\&\qquad \left. - \mathbb {E}\left[ \Vert \lambda \mathbf {M} \varvec{\epsilon }\Vert _{\mathcal {I}{(\varvec{\theta }_0)}}^2 \alpha (\mathbf {Z}, \mathbf {Z} + \lambda \mathbf {M} \varvec{\epsilon })\right] \right| \\&\quad \le \left| \mathbb {E}\left[ \Vert \lambda \mathbf {M}_n \varvec{\epsilon }\Vert _{\mathcal {I}{(\hat{\varvec{\theta }}_n)}}^2 \alpha _n(\mathbf {Z}_n, \mathbf {Z}_n + \lambda \mathbf {M}_n \varvec{\epsilon })\right] \right. \\&\qquad \left. - \mathbb {E}\left[ \Vert \lambda \mathbf {M}\varvec{\epsilon }\Vert _{\mathcal {I}{(\varvec{\theta }_0)}}^2 \alpha _n(\mathbf {Z}_n, \mathbf {Z}_n + \lambda \mathbf {M}\varvec{\epsilon })\right] \right| \\&\qquad + \left| \mathbb {E}\left[ \Vert \lambda \mathbf {M}\varvec{\epsilon }\Vert _{\mathcal {I}{(\varvec{\theta }_0)}}^2 \alpha _n(\mathbf {Z}_n, \mathbf {Z}_n + \lambda \mathbf {M}\varvec{\epsilon })\right] \right. \\&\qquad \left. - \mathbb {E}\left[ \Vert \lambda \mathbf {M} \varvec{\epsilon }\Vert _{\mathcal {I}{(\varvec{\theta }_0)}}^2 \alpha (\mathbf {Z}, \mathbf {Z} + \lambda \mathbf {M} \varvec{\epsilon })\right] \right| . \end{aligned}$$

The first absolute value on the RHS vanishes by assumption. We now prove that the second absolute value on the RHS vanishes. We have

$$\begin{aligned}&\left| \mathbb {E}\left[ \Vert \lambda \mathbf {M} \varvec{\epsilon }\Vert _{\mathcal {I}{(\varvec{\theta }_0)}}^2 \alpha _n(\mathbf {Z}_n, \mathbf {Z}_n + \lambda \mathbf {M} \varvec{\epsilon })\right] \right. \\&\qquad \left. - \mathbb {E}\left[ \Vert \lambda \mathbf {M} \varvec{\epsilon }\Vert _{\mathcal {I}{(\varvec{\theta }_0)}}^2 \alpha (\mathbf {Z}, \mathbf {Z} + \lambda \mathbf {M} \varvec{\epsilon })\right] \right| \\&\quad = \left| \iint \Vert \lambda \mathbf {M} \varvec{\epsilon }\Vert _{\mathcal {I}{(\varvec{\theta }_0)}}^2 \min \{\pi _{\mathbf {Z}_n}(\mathbf {z}), \pi _{\mathbf {Z}_n}(\mathbf {z}\right. \\&\qquad \left. + \lambda \mathbf {M} \varvec{\epsilon })\} \, \varphi (\mathrm {d}\varvec{\epsilon }; \mathbf {0}, \mathbf {1}) \, \mathrm {d}\mathbf {z} \right. \\&\qquad \left. - \iint \Vert \lambda \mathbf {M} \varvec{\epsilon }\Vert _{\mathcal {I}{(\varvec{\theta }_0)}}^2 \min \{\varphi (\mathbf {z}; \mathbf {0}, \mathcal {I}{(\varvec{\theta }_0)}^{-1}),\right. \\&\qquad \left. \times \varphi (\mathbf {z}+ \lambda \mathbf {M} \varvec{\epsilon }; \mathbf {0}, \mathcal {I}{(\varvec{\theta }_0)}^{-1})\} \, \varphi (\mathrm {d}\varvec{\epsilon }; \mathbf {0}, \mathbf {1}) \, \mathrm {d}\mathbf {z} \right| \\&\quad \le \iint \Vert \lambda \mathbf {M} \varvec{\epsilon }\Vert _{\mathcal {I}{(\varvec{\theta }_0)}}^2\\&\left| \min \{\pi _{\mathbf {Z}_n}(\mathbf {z}), \pi _{\mathbf {Z}_n}(\mathbf {z} + \lambda \mathbf {M} \varvec{\epsilon })\} \right. \\&\qquad \left. - \min \{\varphi (\mathbf {z}; \mathbf {0}, \mathcal {I}{(\varvec{\theta }_0)}^{-1}), \varphi (\mathbf {z}+ \lambda \mathbf {M} \varvec{\epsilon }; \mathbf {0}, \mathcal {I}{(\varvec{\theta }_0)}^{-1})\} \right| \,\\&\qquad \times \varphi (\mathrm {d}\varvec{\epsilon }; \mathbf {0}, \mathbf {1}) \, \mathrm {d}\mathbf {z} \\&\quad \le \iint \Vert \lambda \mathbf {M} \varvec{\epsilon }\Vert _{\mathcal {I}{(\varvec{\theta }_0)}}^2\\&\left| \pi _{\mathbf {Z}_n}(\mathbf {z}) - \varphi (\mathbf {z}; \mathbf {0}, \mathcal {I}{(\varvec{\theta }_0)}^{-1})\right| \, \varphi (\mathrm {d}\varvec{\epsilon }; \mathbf {0}, \mathbf {1}) \, \mathrm {d}\mathbf {z} \\&\qquad + \iint \Vert \lambda \mathbf {M} \varvec{\epsilon }\Vert _{\mathcal {I}{(\varvec{\theta }_0)}}^2 \left| \pi _{\mathbf {Z}_n}(\mathbf {z} + \lambda \mathbf {M} \varvec{\epsilon }) \right. \\&\qquad \left. -\, \varphi (\mathbf {z}+ \lambda \mathbf {M} \varvec{\epsilon }; \mathbf {0}, \mathcal {I}{(\varvec{\theta }_0)}^{-1})\right| \, \varphi (\mathrm {d}\varvec{\epsilon }; \mathbf {0}, \mathbf {1}) \, \mathrm {d}\mathbf {z}, \end{aligned}$$

using Jensen’s inequality and $|\min \{a, b\} - \min \{c, d\}| \le |a - c| + |b - d|$ for any real numbers *a*, *b*, *c* and *d*.

The first integral on the RHS vanishes for the same reasons we have seen before (recall (7)). We rewrite the second one as:

8 $$\begin{aligned}&\iint \Vert \mathbf {z}' - \mathbf {z}\Vert _{\mathcal {I}{(\varvec{\theta }_0)}}^2 \left| \pi _{\mathbf {Z}_n}(\mathbf {z}') - \varphi (\mathbf {z}'; \mathbf {0}, \mathcal {I}{(\varvec{\theta }_0)}^{-1})\right| \nonumber \\&\qquad \times \varphi (\mathrm {d}\mathbf {z}'; \mathbf {z}, \lambda ^2 \mathbf {M} \mathbf {M}^T ) \, \mathrm {d}\mathbf {z} \nonumber \\&\quad = \iint \Vert \lambda \varvec{\epsilon }\Vert ^2 \left| \pi _{\mathbf {Z}_n}(\mathbf {z}') - \varphi (\mathbf {z}'; \mathbf {0}, \mathcal {I}{(\varvec{\theta }_0)}^{-1})\right| \, \varphi (\mathrm {d}\varvec{\epsilon }; \mathbf {0}, \mathbf {1}) \, \mathrm {d}\mathbf {z}', \end{aligned}$$

using that $\varphi (\mathrm {d}\mathbf {z}'; \mathbf {z}, \lambda ^2 \mathbf {M} \mathbf {M}^T ) \, \mathrm {d}\mathbf {z} = \varphi (\mathrm {d}\mathbf {z}; \mathbf {z}', \lambda ^2 \mathbf {M} \mathbf {M}^T ) \, \mathrm {d}\mathbf {z}'$ and a change of variables $\varvec{\epsilon }= (\lambda \mathbf {M})^{-1}(\mathbf {z}- \mathbf {z}')$. The last integral vanishes as seen before (recall (7)).

We finish the proof by showing that the assumption

$$\begin{aligned}&\left| \mathbb {E}\left[ \Vert \lambda \mathbf {M}_n \varvec{\epsilon }\Vert _{\mathcal {I}{(\hat{\varvec{\theta }}_n)}}^2 \alpha _n(\mathbf {Z}_n, \mathbf {Z}_n + \lambda \mathbf {M}_n \varvec{\epsilon })\right] \right. \\&\qquad \left. - \mathbb {E}\left[ \Vert \lambda \mathbf {M}\varvec{\epsilon }\Vert _{\mathcal {I}{(\varvec{\theta }_0)}}^2 \alpha _n(\mathbf {Z}_n, \mathbf {Z}_n + \lambda \mathbf {M}\varvec{\epsilon })\right] \right| \rightarrow 0, \end{aligned}$$

is verified when $\mathbf {M}_n \mathbf {M}_n^T = \mathcal {I}{(\hat{\varvec{\theta }}_n)}^{-1} \rightarrow \mathbf {M}\mathbf {M}^T = \mathcal {I}{(\varvec{\theta }_0)}^{-1}$. In this case,

$$\begin{aligned}&\left| \mathbb {E}\left[ \Vert \lambda \mathbf {M}_n \varvec{\epsilon }\Vert _{\mathcal {I}{(\hat{\varvec{\theta }}_n)}}^2 \alpha _n(\mathbf {Z}_n, \mathbf {Z}_n + \lambda \mathbf {M}_n \varvec{\epsilon })\right] \right. \\&\qquad \left. - \mathbb {E}\left[ \Vert \lambda \mathbf {M}\varvec{\epsilon }\Vert _{\mathcal {I}{(\varvec{\theta }_0)}}^2 \alpha _n(\mathbf {Z}_n, \mathbf {Z}_n + \lambda \mathbf {M}\varvec{\epsilon })\right] \right| \\&\quad = \left| \mathbb {E}\left[ \Vert \lambda \varvec{\epsilon }\Vert ^2 \alpha _n(\mathbf {Z}_n, \mathbf {Z}_n + \lambda \mathbf {M}_n \varvec{\epsilon })\right] \right. \\&\qquad \left. - \mathbb {E}\left[ \Vert \lambda \varvec{\epsilon }\Vert ^2 \alpha _n(\mathbf {Z}_n, \mathbf {Z}_n + \lambda \mathbf {M}\varvec{\epsilon })\right] \right| \\&\quad = \left| \iint \Vert \lambda \varvec{\epsilon }\Vert ^2 \min \{\pi _{\mathbf {Z}_n}(\mathbf {z}), \pi _{\mathbf {Z}_n}(\mathbf {z} \right. \\&\qquad \left. + \lambda \mathbf {M}_n \varvec{\epsilon })\} \, \varphi (\mathrm {d}\varvec{\epsilon }; \mathbf {0}, \mathbf {1}) \, \mathrm {d}\mathbf {z} \right. \\&\qquad - \left. \iint \Vert \lambda \varvec{\epsilon }\Vert ^2 \min \{\pi _{\mathbf {Z}_n}(\mathbf {z}), \pi _{\mathbf {Z}_n}(\mathbf {z} + \lambda \mathbf {M} \varvec{\epsilon })\} \,\right. \\&\left. \varphi (\mathrm {d}\varvec{\epsilon }; \mathbf {0}, \mathbf {1}) \, \mathrm {d}\mathbf {z} \right| \\&\quad \le \iint \Vert \lambda \varvec{\epsilon }\Vert ^2 \left| \min \{\pi _{\mathbf {Z}_n}(\mathbf {z}), \pi _{\mathbf {Z}_n}(\mathbf {z} + \lambda \mathbf {M}_n \varvec{\epsilon })\} \right. \\&\qquad \left. - \min \{\pi _{\mathbf {Z}_n}(\mathbf {z}), \pi _{\mathbf {Z}_n}(\mathbf {z} + \lambda \mathbf {M} \varvec{\epsilon })\} \right| \, \varphi (\mathrm {d}\varvec{\epsilon }; \mathbf {0}, \mathbf {1}) \, \mathrm {d}\mathbf {z} \\&\quad \le \iint \Vert \lambda \varvec{\epsilon }\Vert ^2 \left| \pi _{\mathbf {Z}_n}(\mathbf {z} + \lambda \mathbf {M}_n \varvec{\epsilon }) \right. \\&\qquad \left. - \pi _{\mathbf {Z}_n}(\mathbf {z} + \lambda \mathbf {M} \varvec{\epsilon }) \right| \, \varphi (\mathrm {d}\varvec{\epsilon }; \mathbf {0}, \mathbf {1}) \, \mathrm {d}\mathbf {z}, \end{aligned}$$

using Jensen’s inequality and $|\min \{a, b\} - \min \{c, d\}| \le |a - c| + |b - d|$ for any real numbers *a*, *b*, *c* and *d*.

Now, using the triangle inequality,

9 $$\begin{aligned}&\iint \Vert \lambda \varvec{\epsilon }\Vert ^2 \left| \pi _{\mathbf {Z}_n}(\mathbf {z} + \lambda \mathbf {M}_n \varvec{\epsilon }) \right. \nonumber \\&\qquad \left. - \pi _{\mathbf {Z}_n}(\mathbf {z} + \lambda \mathbf {M} \varvec{\epsilon }) \right| \, \varphi (\mathrm {d}\varvec{\epsilon }; \mathbf {0}, \mathbf {1}) \, \mathrm {d}\mathbf {z} \nonumber \\&\quad \le \iint \Vert \lambda \varvec{\epsilon }\Vert ^2 \left| \pi _{\mathbf {Z}_n}(\mathbf {z} + \lambda \mathbf {M}_n \varvec{\epsilon }) \right. \nonumber \\&\qquad \left. - \varphi (\mathbf {z}+ \lambda \mathbf {M}_n \varvec{\epsilon }; \mathbf {0}, \mathcal {I}{(\varvec{\theta }_0)}^{-1}) \right| \, \varphi (\mathrm {d}\varvec{\epsilon }; \mathbf {0}, \mathbf {1}) \, \mathrm {d}\mathbf {z} \nonumber \\&\qquad + \iint \Vert \lambda \varvec{\epsilon }\Vert ^2 \left| \varphi (\mathbf {z}+ \lambda \mathbf {M}_n \varvec{\epsilon }; \mathbf {0}, \mathcal {I}{(\varvec{\theta }_0)}^{-1}) - \varphi (\mathbf {z}\right. \nonumber \\&\qquad \left. + \lambda \mathbf {M} \varvec{\epsilon }; \mathbf {0}, \mathcal {I}{(\varvec{\theta }_0)}^{-1})\right| \, \varphi (\mathrm {d}\varvec{\epsilon }; \mathbf {0}, \mathbf {1}) \, \mathrm {d}\mathbf {z} \nonumber \\&\qquad + \iint \Vert \lambda \varvec{\epsilon }\Vert ^2 \left| \varphi (\mathbf {z}+ \lambda \mathbf {M} \varvec{\epsilon }; \mathbf {0}, \mathcal {I}{(\varvec{\theta }_0)}^{-1}) \right. \nonumber \\&\qquad \left. - \pi _{\mathbf {Z}_n}(\mathbf {z} + \lambda \mathbf {M} \varvec{\epsilon })\right| \, \varphi (\mathrm {d}\varvec{\epsilon }; \mathbf {0}, \mathbf {1}) \, \mathrm {d}\mathbf {z}. \end{aligned}$$

We now prove that each of the integrals on the RHS vanishes. We start with the first one,

$$\begin{aligned}&\iint \Vert \lambda \varvec{\epsilon }\Vert ^2 \left| \pi _{\mathbf {Z}_n}(\mathbf {z} + \lambda \mathbf {M}_n \varvec{\epsilon })\right. \\&\qquad \left. - \varphi (\mathbf {z}+ \lambda \mathbf {M}_n \varvec{\epsilon }; \mathbf {0}, \mathcal {I}{(\varvec{\theta }_0)}^{-1}) \right| \, \varphi (\mathrm {d}\varvec{\epsilon }; \mathbf {0}, \mathbf {1}) \, \mathrm {d}\mathbf {z} \\&\quad = \iint \Vert \mathbf {z}' - \mathbf {z}\Vert _{\mathcal {I}{(\hat{\varvec{\theta }}_n)}}^2 \left| \pi _{\mathbf {Z}_n}(\mathbf {z}') - \varphi (\mathbf {z}'; \mathbf {0}, \mathcal {I}{(\varvec{\theta }_0)}^{-1}) \right| \,\\&\varphi (\mathrm {d}\mathbf {z}'; \mathbf {z}, \lambda ^2 \mathbf {M}_n \mathbf {M}_n^T) \, \mathrm {d}\mathbf {z}, \end{aligned}$$

using the change of variable $\mathbf {z}' = \mathbf {z} + \lambda \mathbf {M}_n \varvec{\epsilon }$. As we have seen before, the last integral vanishes (recall (8)). The third integral on the RHS in (9) vanishes for similar reasons.

For the second one, we use that $\mathbf {M}_n \rightarrow \mathbf {M}$ in $\mathbb {P}^\mathbf {Y}$-probability. This is true because $\mathbf {M}_n \mathbf {M}_n^T \rightarrow \mathbf {M} \mathbf {M}^T$ in $\mathbb {P}^\mathbf {Y}$-probability and the Cholesky decomposition yields a continuous map. Now, using Devroye et al., (2018, Proposition 2.1) and Cauchy–Schwarz inequality,

$$\begin{aligned}&\iint \Vert \lambda \varvec{\epsilon }\Vert ^2 \left| \varphi (\mathbf {z}; -\lambda \mathbf {M}_n \varvec{\epsilon }, \mathcal {I}{(\varvec{\theta }_0)}^{-1}) - \varphi (\mathbf {z}; -\lambda \mathbf {M} \varvec{\epsilon }, \mathcal {I}{(\varvec{\theta }_0)}^{-1})\right| \,\\&\varphi (\mathrm {d}\varvec{\epsilon }; \mathbf {0}, \mathbf {1}) \, \mathrm {d}\mathbf {z} \\&\quad \le \int \Vert \lambda \varvec{\epsilon }\Vert ^2 \lambda \left[ \varvec{\epsilon }^T (\mathbf {M}^{-1} \mathbf {M}_n - \mathbf {1})^T (\mathbf {M}^{-1} \mathbf {M}_n - \mathbf {1})\varvec{\epsilon }\right] ^{1/2} \,\\&\varphi (\mathrm {d}\varvec{\epsilon }; \mathbf {0}, \mathbf {1}) \\&\quad \le \lambda ^3 \left[ \int \Vert \epsilon \Vert ^4 \, \varphi (\mathrm {d}\varvec{\epsilon }; \mathbf {0}, \mathbf {1})\right] ^{1/2} \left[ \int \varvec{\epsilon }^T (\mathbf {M}^{-1} \mathbf {M}_n - \mathbf {1})^T (\mathbf {M}^{-1} \mathbf {M}_n \right. \\&\qquad \left. - \mathbf {1})\varvec{\epsilon }\, \varphi (\mathrm {d}\varvec{\epsilon }; \mathbf {0}, \mathbf {1})\right] ^{1/2}. \end{aligned}$$

The first integral on the RHS is bounded. We write the second one as an expectation:

$$\begin{aligned}&\mathbb {E}[\varvec{\epsilon }^T \mathbf {A}_n \varvec{\epsilon }] = \mathbb {E}[\varvec{\epsilon }^T \mathbf {Q}_n \varvec{\varLambda }_n \mathbf {Q}_n^T \varvec{\epsilon }]\\&= \mathbb {E}\left[ \sum _{j = 1}^d \lambda _{j, n} \xi _{j, n}^2\right] = \sum _{j = 1}^d \lambda _{j, n} = \text {tr}(\mathbf {A}_n) \rightarrow 0, \end{aligned}$$

using an eigendecomposition of $\mathbf {A}_n$ and that $\varvec{\xi }_n := (\xi _{1, n}, \ldots , \xi _{d, n})^T := \mathbf {Q}_n^T \varvec{\epsilon }$ is a random vector with independent standard normal components, where $\mathbf {A}_n := (\mathbf {M}^{-1} \mathbf {M}_n - \mathbf {1})^T (\mathbf {M}^{-1} \mathbf {M}_n - \mathbf {1})$, $\mathbf {Q}_n$ is an orthogonal matrix whose columns are the eigenvectors of $\mathbf {A}_n$, and $\varvec{\varLambda }_n$ is a diagonal matrix whose entries $\lambda _{1, n}, \ldots , \lambda _{d, n}$ are the eigenvalues of $\mathbf {A}_n$. This concludes the proof. $\square$

### Proof (Corollary 1)

We first denote $\mathbf {S} := \lambda \mathbf {M}$ and thus note that $\mathbf {Z}' = \mathbf {Z} + \mathbf {S}\varvec{\epsilon }$, where $\mathbf {Z} \sim \varphi (\, \cdot \,; \mathbf {0}, \mathcal {I}{(\varvec{\theta }_0)}^{-1})$ and $\varvec{\epsilon }\sim \varphi (\, \cdot \,; \mathbf {0}, \mathbf {1})$. We have

$$\begin{aligned}&{\text {ESJD}}(\lambda , \mathbf {M}) \\&\quad = \mathbb {E}\left[ \Vert \mathbf {S}\varvec{\epsilon }\Vert _{\mathcal {I}{(\varvec{\theta }_0)}}^2 \min \left\{ 1, \frac{\varphi (\mathbf {Z} + \mathbf {S}\varvec{\epsilon }; \mathbf {0}, \mathcal {I}{(\varvec{\theta }_0)}^{-1})}{\varphi (\mathbf {Z}; \mathbf {0}, \mathcal {I}{(\varvec{\theta }_0)}^{-1})} \right\} \right] \\&\quad = \mathbb {E}\bigg [\Vert \mathbf {S}\varvec{\epsilon }\Vert _{\mathcal {I}{(\varvec{\theta }_0)}}^2 \min \bigg \{1, \exp \bigg (- \frac{1}{2}\varvec{\epsilon }^T \mathbf {S}^T \mathcal {I}{(\varvec{\theta }_0)} \, \mathbf {S} \varvec{\epsilon }\\&\qquad + \mathbf {Z}^T \mathbf {S}^T \mathcal {I}{(\varvec{\theta }_0)} \, \varvec{\epsilon }\bigg )\bigg \} \bigg ] \\&\quad = \mathbb {E}\bigg [\Vert \mathbf {S}\varvec{\epsilon }\Vert _{\mathcal {I}{(\varvec{\theta }_0)}}^2 \mathbb {E}\bigg [\min \bigg \{1, \exp \bigg (- \frac{1}{2}\varvec{\epsilon }^T \mathbf {S}^T \mathcal {I}{(\varvec{\theta }_0)} \, \mathbf {S} \varvec{\epsilon }\\&\qquad + \mathbf {Z}^T \mathbf {S}^T \mathcal {I}{(\varvec{\theta }_0)} \, \varvec{\epsilon }\bigg )\bigg \} \mid \varvec{\epsilon }\bigg ] \bigg ]. \end{aligned}$$

In the following, we make use of the fact that for a univariate normal random variable *X* with $X \sim \varphi (\,\cdot \,; m, s^2)$, we have that

$$\begin{aligned} \mathbb {E}\left[ \min \{1, \exp (X)\}\right] = \varPhi \left( \frac{m}{s}\right) + \exp \left( m + \frac{s^2}{2}\right) \, \varPhi \left( -s - \frac{m}{s}\right) . \end{aligned}$$

In particular, if $m=-s^2/2$,

10 $$\begin{aligned} \mathbb {E}\left[ \min \{1, \exp (X)\}\right] = 2\varPhi \left( -\frac{s}{2}\right) . \end{aligned}$$

Consider the case where $\mathbf {M} = \mathbf {1}$. Thus, given $\varvec{\epsilon }$, $- \frac{1}{2}\varvec{\epsilon }^T \mathbf {S}^T \mathcal {I}{(\varvec{\theta }_0)} \, \mathbf {S} \varvec{\epsilon } + \mathbf {Z}^T \mathbf {S}^T \mathcal {I}{(\varvec{\theta }_0)} \, \varvec{\epsilon } = - \frac{\lambda ^2}{2}\varvec{\epsilon }^T \mathcal {I}{(\varvec{\theta }_0)} \, \varvec{\epsilon } + \lambda \mathbf {Z}^T \mathcal {I}{(\varvec{\theta }_0)} \, \varvec{\epsilon }$ is a Gaussian random variable with $m = - \frac{\lambda ^2}{2}\varvec{\epsilon }^T \mathcal {I}{(\varvec{\theta }_0)} \, \varvec{\epsilon }$ and $s^2 = \lambda ^2 \varvec{\epsilon }^T \mathcal {I}{(\varvec{\theta }_0)} \, \varvec{\epsilon }$, implying that

$$\begin{aligned}&\mathbb {E}\bigg [\Vert \mathbf {S}\varvec{\epsilon }\Vert _{\mathcal {I}{(\varvec{\theta }_0)}}^2 \mathbb {E}\bigg [\min \bigg \{1, \exp \bigg (- \frac{1}{2}\varvec{\epsilon }^T \mathbf {S}^T \mathcal {I}{(\varvec{\theta }_0)} \, \mathbf {S} \varvec{\epsilon } \\&\qquad + \mathbf {Z}^T \mathbf {S}^T \mathcal {I}{(\varvec{\theta }_0)} \, \varvec{\epsilon }\bigg )\bigg \} \mid \varvec{\epsilon }\bigg ] \bigg ] \\&\quad = 2 \lambda ^2 \mathbb {E}\left[ \Vert \varvec{\epsilon }\Vert _{\mathcal {I}{(\varvec{\theta }_0)}}^2 \varPhi \left( -\lambda \, \frac{\Vert \varvec{\epsilon }\Vert _{\mathcal {I}{(\varvec{\theta }_0)}}}{2}\right) \right] . \end{aligned}$$

The formulae for $\mathrm {ESJD}$ with $\mathbf {M}$ such that $\mathbf {M}\mathbf {M}^T = \mathcal {I}_{\varvec{\theta }_0}^{-1}$ and the expected acceptance probabilities are derived analogously. $\square$

### Proof (Proposition 1)

The result follows directly from a result in the convex order literature, stating that, for any $d \ge 2$ exchangeable random variables $X_1, \ldots , X_d$ and any convex function $\phi$, we have

$$\begin{aligned} \mathbb {E}\left[ \phi \left( \frac{1}{d} \sum _{i=1}^d X_i\right) \right] \le \mathbb {E}\left[ \phi \left( \frac{1}{d - 1} \sum _{i=1}^{d - 1} X_i\right) \right] , \end{aligned}$$

whenever the expectations exist (Müller and Stoyan 2002, Corollary 1.5.24). We are thus able to conclude by setting $X_i = \epsilon _i^2$ and $\phi (x) = \varPhi \left( -(\ell / 2) \surd {x}\right)$ for all $x \ge 0$ given that this function is convex. $\square$

### Proof (Proposition 2)

We prove that

$$\begin{aligned}&2\lambda ^2 \, \mathbb {E}\left\{ \Vert \varvec{\epsilon }\Vert ^2 \, \varPhi \left( -\lambda \, \frac{\Vert \varvec{\epsilon }\Vert }{2}\right) \right\} = 2\ell ^2 \, \\&\mathbb {E}\left\{ \frac{\Vert \varvec{\epsilon }\Vert ^2}{d} \, \varPhi \left( -\ell \, \frac{\Vert \varvec{\epsilon }\Vert /\surd {d}}{2}\right) \right\} \rightarrow 2\ell ^2 \, \varPhi \left( - \frac{\ell }{2}\right) . \end{aligned}$$

The convergence

$$\begin{aligned} 2 \, \mathbb {E}\left\{ \varPhi \left( -\lambda \, \frac{\Vert \varvec{\epsilon }\Vert }{2}\right) \right\}&= 2 \, \mathbb {E}\left\{ \varPhi \left( -\ell \, \frac{\Vert \varvec{\epsilon }\Vert /\surd {d}}{2}\right) \right\} \\&\rightarrow 2 \, \varPhi \left( - \frac{\ell }{2}\right) \end{aligned}$$

follows using similar arguments.

By the strong law of large numbers, we have that $\Vert \varvec{\epsilon }\Vert ^2 / d \rightarrow 1$ almost surely, and then

$$\begin{aligned} \frac{\Vert \varvec{\epsilon }\Vert ^2}{d} \, \varPhi \left( -\ell \, \frac{\Vert \varvec{\epsilon }\Vert /\surd {d}}{2}\right) \rightarrow \varPhi \left( - \frac{\ell }{2}\right) , \end{aligned}$$

almost surely. To prove that the expectation converges, we show that

$$\begin{aligned} \frac{\Vert \varvec{\epsilon }\Vert ^2}{d} \, \varPhi \left( -\ell \, \frac{\Vert \varvec{\epsilon }\Vert /\surd {d}}{2}\right) \end{aligned}$$

is uniformly integrable. To prove this, we show that

$$\begin{aligned} \sup _d \mathbb {E}\left\{ \left( \frac{\Vert \varvec{\epsilon }\Vert ^2}{d} \, \varPhi \left( -\ell \, \frac{\Vert \varvec{\epsilon }\Vert /\surd {d}}{2}\right) \right) ^2 \right\} < \infty . \end{aligned}$$

Using that $0 \le \varPhi \le 1$ and that $\Vert \varvec{\epsilon }\Vert ^2$ has a chi-square distribution with *d* degrees of freedom,

$$\begin{aligned} \mathbb {E}\left\{ \left( \frac{\Vert \varvec{\epsilon }\Vert ^2}{d} \, \varPhi \left( -\ell \, \frac{\Vert \varvec{\epsilon }\Vert /\surd {d}}{2}\right) \right) ^2 \right\}&\le \mathbb {E}\left\{ \left( \frac{\Vert \varvec{\epsilon }\Vert ^2}{d} \, \right) ^2 \right\} \\&= \frac{2d + d^2}{d^2}, \end{aligned}$$

which has a finite supremum. This concludes the proof that

$$\begin{aligned} 2\ell ^2 \, \mathbb {E}\left\{ \frac{\Vert \varvec{\epsilon }\Vert ^2}{d} \, \varPhi \left( -\ell \, \frac{\Vert \varvec{\epsilon }\Vert /\surd {d}}{2}\right) \right\} \rightarrow 2\ell ^2 \, \varPhi \left( - \frac{\ell }{2}\right) . \end{aligned}$$

The function $2\ell ^2 \, \varPhi \left( - \frac{\ell }{2}\right)$ can be optimized numerically and is maximized by $\ell = \hat{\ell } := 2.38$. $\square$

## Footnotes

1 See ancillary files on https://arxiv.org/abs/2104.06384.
